# Mesothelin- and nucleolin-specific T cells from combined short peptides effectively kill triple-negative breast cancer cells

**DOI:** 10.1186/s12916-024-03625-3

**Published:** 2024-09-18

**Authors:** Suyanee Thongchot, Krittaya Aksonnam, Jaturawitt Prasopsiri, Malee Warnnissorn, Doonyapat Sa-nguanraksa, Pornchai O-Charoenrat, Peti Thuwajit, Pa-thai Yenchitsomanus, Chanitra Thuwajit

**Affiliations:** 1 Department of Immunology, Faculty of Medicine Siriraj Hospital, Mahidol University, Bangkok, 10700 Thailand; 2 Siriraj Center of Research Excellence for Cancer Immunotherapy (SiCORE-CIT), Research Department, Faculty of Medicine Siriraj Hospital, Mahidol University, Bangkok, 10700 Thailand; 3 Department of Pathology, Faculty of Medicine Siriraj Hospital, Mahidol University, Bangkok, 10700 Thailand; 4 Division of Head Neck and Breast Surgery, Department of Surgery, Faculty of Medicine Siriraj Hospital, Mahidol University, Bangkok, 10700 Thailand; 5 Breast Center, MedPark Hospital, Bangkok, 10110 Thailand; 6 Division of Molecular Medicine, Research Department, Faculty of Medicine Siriraj Hospital, Mahidol University, Bangkok, 10700 Thailand

**Keywords:** Mesothelin, Nucleolin, Short peptide, Triple-negative breast cancer, Immunotherapy

## Abstract

**Background:**

Triple-negative breast cancer (TNBC), known for its aggressiveness and limited treatment options, presents a significant challenge. Adoptive cell transfer, involving the ex vivo generation of antigen-specific T cells from peripheral blood mononuclear cells (PBMCs), emerges as a promising approach. The overexpression of mesothelin (MSLN) and nucleolin (NCL) in TNBC samples underscores their potential as targets for T cell therapy. This study explored the efficacy of multi-peptide pulsing of PBMCs to generate MSLN/NCL-specific T cells targeting MSLN^+^/NCL^+^ TNBC cells.

**Methods:**

TNBC patient samples were confirmed for both MSLN and NCL expression via immunohistochemistry. Synthesized MSLN and NCL peptides were combined and administered to activate PBMCs from healthy donors. The cancer-killing ability of the resultant T cells was assessed using crystal violet staining, and their subtypes and cytotoxic cytokines were characterized through flow cytometry and cytokine bead array.

**Results:**

Findings showed that 85.3% (127/149) of TNBC cases were positive for either MSLN or NCL, or both; with single positivity rates for MSLN and NCL of 14.1% and 28.9%, respectively. MSLN and NCL peptides, with high binding affinity for HLA-A*02, were combined and introduced to activated PBMCs from healthy donors. The co-pulsed PBMCs significantly induced T_EM_ and T_EMRA_ CD3^+^/CD8^+^ T cells and IFN-γ production, compared to single-peptide pulsed or unpulsed conditions. Notably, MSLN/NCL-specific T cells successfully induced cell death in MSLN^+^/NCL^+^ MDA-MB-231 cells, releasing key cytotoxic factors such as perforin, granzymes A and B, Fas ligand, IFN-γ, and granulysin.

**Conclusions:**

These findings serve as a proof-of-concept for using multiple immunogenic peptides as a novel therapeutic approach in TNBC patients.

**Supplementary Information:**

The online version contains supplementary material available at 10.1186/s12916-024-03625-3.

## Background

According to the Global Burden of Cancer Study, female breast cancer ascended to the top of global cancer incidence in 2020, accounting for 11.7% of all cancer diagnoses and surpassing lung cancer [1]. Triple-negative breast cancer (TNBC), a particularly aggressive subtype, carries the most dismal prognosis and has limited therapeutic avenues [2]. TNBC patients are ineligible for hormone receptor or targeted therapeutic interventions, attributed to the absence of estrogen receptor, progesterone receptor, and human epidermal growth factor receptor 2 expression. Presently, chemotherapy, either as a standalone treatment or in conjunction with surgery and/or radiotherapy, represents the predominant systemic therapy for TNBC [3]. Nevertheless, chemoresistance, recurrence, and metastasis subsequent to chemotherapy and radiation persist as significant impediments to the survival of TNBC patients [4]. Thus, the identification and development of new potential therapeutic strategies and biological targets are urgent.

The heightened immune cell infiltration, noticeable programmed death-ligand 1 (PD-L1) expression, and an abundance of nonsynonymous mutations render TNBC more amenable to immunotherapy than other breast cancer subtypes [5]. Cancer cells possess a myriad of evasion tactics to circumvent detection and elimination by the immune system, one significant strategy being the expression of immune checkpoints that attenuate immune cell activity. Consequently, the administration of immune checkpoint inhibitors (ICIs), such as anti-PD-L1 or anti-programmed death-1 (PD-1), has been shown to activate antitumor T cell responses [6]. Currently, ICI treatment has been deployed in managing TNBC patients, although the efficacy of ICI monotherapy remains suboptimal [7, 8]. Adoptive cell immunotherapy, characterized by the ex vivo expansion of antigen-specific T cells, presents a promising therapeutic avenue due to its specificity, capacity for self-replication, and minimal toxicity [9]. To generate antigen-specific T cells, various methodologies have been investigated, including the transfection of dendritic cells with DNA constructs encoding tumor antigen epitopes [10], the pulsing of dendritic cells with antigen peptides [11], as well as the stimulation of peripheral blood mononuclear cells (PBMCs) with synthetic tumor-associated antigen (TAA) or tumor-specific antigen (TSA) peptides [12, 13].

Recent reports have disclosed the successful generation of multi-peptide-specific T cells through the stimulation of PBMCs with multi-peptides in the context of hematopoietic malignancies [13–15]. These multi-peptide-specific T cells, stimulated by multi-peptides, resulted in an overexpression of TAAs in multiple myeloma, and they were capable of producing interferon-gamma (IFN-γ), granzyme B, and perforin, serving as surrogates of cytolytic activity [13]. Furthermore, the multi-peptide-specific T cells exhibited cytotoxic capabilities against targeted leukemia cells [14]. The results also demonstrated the safety profile of multi-peptide-specific T cells in patients with multiple myeloma, alongside evidence demonstrating clinical benefits concurrent with the in vivo expansion of tumor-specific T cells [14, 15]. Additionally, multi-peptide-specific T cells generated from the peripheral blood of patients with metastatic or locally recurrent breast cancer across all subtypes were evaluated in a human trial [16]. These multi-peptide-specific T cells were notably persistent, inducing disease stabilization and antigen spreading in the case of a patient with refractory breast cancer [16]. Collectively, these findings suggest that the simultaneous targeting of multiple antigens may mitigate the risk of tumor immune evasion, thereby enhancing the utility of adoptive T cell therapy.

Several TAAs have been identified as potential targets for TNBC therapy, notably mesothelin (MSLN) and nucleolin (NCL), which have been reported to hold promise in this context [17–21]. MSLN, a membrane-bound protein, is sparingly expressed in normal cells but is markedly overexpressed in various cancer cells, including TNBC [18, 22]. Studies have indicated a prevalence of MSLN expression in 34%–67% of TNBC samples, associating it with cancer cell proliferation and tumor progression [23, 24]. NCL, on the other hand, demonstrated overexpression in 75%–80% of TNBC cases [20, 25], predominantly localized to the cell membrane of various cancer cells. NCL functions as a receptor for numerous oncogenic ligands and plays a pivotal role in various processes of oncogenesis, correlating with poorer overall survival and disease-free survival rates [26, 27]. The restricted expression of MSLN and NCL in normal cells, juxtaposed with their overexpression in TNBC, positions them as appealing candidates for constructing antigen-specific T cells for adoptive T cell therapy. Our research group has previously reported on the generation of MSLN-specific T cells and NCL-specific T cells through the stimulation with self-differentiated myeloid-derived antigen-presenting cells reactive against tumor expressing either MSLN-derived dendritic cells (MSLN-SmartDCs) or NCL-derived dendritic cells (NCL-SmartDCs) [20, 28]. These MSLN-specific T cells and NCL-specific T cells were observed to exhibit potent effective killing with cytotoxic activity against TNBC cells [20, 28]. Nonetheless, given TNBC’s heterogeneity, T cells targeting a singular antigen may not suffice to eliminate all TNBC cells effectively.

In an effort to surmount the limitations imposed by the heterogeneity of TNBC, the present study was designed to generate MSLN/NCL-specific T cells through concurrent stimulation of PBMCs with a combination of MSLNp and NCLp. Our focus was on elucidating the subtypes of these MSLN/NCL-specific T cells and evaluating their anti-cancer efficacy. The outcomes of this investigation could substantiate the utility of employing multiple peptides in cancer vaccines and multi-peptide-specific T cells, potentially paving the way for their future application in adoptive T cell therapy for patients with TNBC.

## Methods

### Blood collection

PBMCs were collected from three human leukocyte antigen (HLA)-A*02 healthy donors (HDs) following the provision of written informed consent under the approval. This procedure was conducted in accordance with the protocol approved by the Siriraj Institutional Review Board, Faculty of Medicine Siriraj Hospital, Mahidol University (COA no. Si 580/2018).

### HLA typing

The HLA alleles typing for all cell lines and HDs was conducted using the LABType XR Class I, A, B, and C Locus Typing Kit (RSOX1AT, RSOX1BT, and RSOX1CT; Invitrogen Corporation, Carlsbad, CA) at the Department of Transfusion Medicine, Faculty of Medicine Siriraj Hospital, Mahidol University (Additional file 1: Table S1). Additionally, the expression of HLA on cancer cells was evaluated using CytoFLEX flow cytometry (Beckman Coulter, Brea, CA) and a FITC-labeled goat anti‑HLA-ABC W6/32 monoclonal antibody (14–9983-82; eBioscience Inc. San Diego, CA).

### TNBC clinical samples

Formalin-fixed paraffin-embedded (FFPE) tissue samples from 149 TNBC patients who underwent surgical procedures at Siriraj Hospital were obtained. The clinicopathological data for these patients are maintained by the Department of Pathology, Faculty of Medicine Siriraj Hospital. This recruitment was carried out in compliance with the protocol approved by the institutional committee (COA no. Si 580/2018).

### Cell lines and cell culture

Commercial human TNBC cell line (MDA-MB-231, #HTB-26, HLA-A*02:17), a normal mammary cell line (MCF-10A, #CRL-10317), and HLA-A*02 T2 cells (#CRL-1992) expressing an empty HLA-A*02:01 allele of the MHC class I molecule on the cell surface were acquired from the American Type Culture Collection (ATCC) (Manassas, VA). Additionally, MSLN-MDA-MB-231 cells previously developed by our group were utilized [28]. MDA-MB-231 and MSLN-MDA-MB-231 cells were propagated in Dulbecco’s Modified Eagle Medium (DMEM, Gibco, Thermo Fisher Scientific Inc., Waltham, MA) supplemented with 10% fetal bovine serum (FBS) (v/v) (Gibco), 100 U/ml of penicillin, and 100 µg/ml of streptomycin (Sigma-Aldrich Corporation, St Louis, MO). MCF-10A cells were cultivated in DMEM augmented with 5% horse serum (Invitrogen Corporation), 100 U/ml of penicillin, 100 µg/ml of streptomycin, 20 ng/ml epithelial growth factor (EGF, PeproTech, Cranbury, NJ), 0.5 mg/ml hydrocortisone (Sigma–Aldrich Corporation), 100 ng/ml cholera toxin (Sigma–Aldrich), and 10 μg/ml insulin (Sigma–Aldrich Corporation). HLA-A*02 T2 cells were maintained in RPMI 1640 (Gibco) supplemented with 2 mM of L-glutamine (Gibco) and 20% FBS (v/v) (Gibco). All cell cultures were maintained at 37 °C in a 5% CO_2_ incubator. Cell viability was assessed at each passage using a standard trypsinization protocol, with cells being passaged a maximum of 20 times for experimental purposes. The cells were also screened every 3 months for negative mycoplasma contamination using the mycoplasma nested PCR primer set (BioDesign Co. Ltd., Pathumthani, Thailand) throughout the study.

### NCL siRNA transfection

To establish MDA-MB-231-NCL^KD^ (MSLN^−^/NCL^−^-M231) and MSLN-MDA-MB-231-NCL^KD^ (MSLN^+^/NCL^−^-M231) cell lines, 1 × 10^5^ cells of MDA-MB-231 and MSLN-MDA-MB-231 (Additional file 2: Table S2) were each seeded in a 6-well plate and incubated overnight. Subsequently, 1 nM of si-*NCL* (sc-29230, Santa Cruz Biotechnology Inc, Dallas, Texas) or 1 nM si-control (sc-37007, Santa Cruz Biotechnology Inc) were prepared in Opti-MEM (Gibco) and mixed with Lipofectamine 3000 (Invitrogen Corporation) before being added to the cells. At 24 h post-transfection, the medium containing siRNA was removed and replaced with DMEM supplemented with 10% FBS, and incubated, followed by further incubation at 37 °C for 48 h. Post-incubation, the cells were processed for western blot analysis and subjected to a killing assay.

### Western blot analysis

To evaluate the protein expression of cell lines listed in Additional file 2: Table S2, collected cell pellets were lysed using RIPA buffer (Santa Cruz Biotechnology Inc). The concentration of total protein was determined by the Bradford colorimetric assay (Thermo Scientific, MA). Proteins from cell lysates were separated in 10% sodium dodecyl sulfate–polyacrylamide gel electrophoresis (SDS-PAGE) and subsequently transferred to a polyvinylidene fluoride (PVDF) membrane (GE Healthcare Technologies, Vienna, Austria) with a semi-dry blotter. The membranes were blocked using 5% skim milk diluted in 0.1% Tween 20 in 1X Tris-buffered saline (TBST) for 1 h. The membranes were then incubated with primary antibodies: 1:500 dilution of mouse anti-human MSLN monoclonal antibody (sc-271540, Santa Cruz Biotechnology Inc), 1:500 dilution of rabbit anti-human NCL monoclonal antibody (14,574, Cell Signaling Technology Inc, Beverly, MA 1:5,000 dilution of mouse anti-human β-actin monoclonal antibody (sc-47778, Santa Cruz Biotechnology Inc) as an internal control. Subsequent incubation with 1:2,000 diluted goat anti-mouse or anti-rabbit IgG antibody conjugated with horseradish peroxidase (HRP) was performed. Detection of the signal was carried out using Western Enhanced Chemiluminescence (ECL) Kit (Bio-Rad Laboratories, CA) and photographed using the Gel Doc system (Bio-Rad Laboratories). The expression levels of MSLN and NCL proteins were quantified by normalizing their band intensities to that of the β-actin internal control using ImageJ software version 1.53e (National Institutes of Health, Bethesda, MD).

### Immunohistochemistry

The immunohistochemistry (IHC) analyses previously reported for MSLN [28] and NCL [20] in TNBC samples were re-scored by calculating scores through the multiplication of intensity (I) and the percentage of MSLN or NCL positive cells (P). The intensity was graded as follows: 0 (negative), 1 (weak), 2 (moderate), and 3 (strong), while the percentage of positive cells (P) graded was scored as: 0 (no positive cells), 1 (1%–25%), 2 (26%–50%), 3 (51%–75%), and 4 (76%–100%). The resultant IHC scores ranged from 0 to 12 and facilitated the classification of samples into two groups. An IHC score of 0 was categorized as negative for MSLN expression (MSLN^−^), whereas an IHC score greater than 0 was categorized as positive for MSLN expression group (MSLN^+^) [28]. For determining the expression levels of NCL, cutoffs for low and high expressions were established utilizing the maxstat, survival, survminer, and tidyverse packages in R Studio software, applying log-rank statistics based on the overall survival of TNBC patients. The optimal cutpoint for NCL expression defined by this methodology was 4.67; thus, NCL expression scores ≤ 4.67 were classified as low expression (NCL^Low^), while scores > 4.67 were classified as high expression (NCL^High^).

### Double immunofluorescence staining

To detect the co-expression of MSLN and NCL in TNBC tissues, double immunofluorescence staining was performed. Initially, formalin-fixed paraffin-embedded samples were incubated in 10 mM citrate buffer (pH 6.0) at 95 °C for 1 h. Subsequent blocking involved a 30 min incubation with 3% H_2_O_2_ followed by a 30 min incubation with 10% skim milk at room temperature. The tissues were then stained with anti-MSLN antibody (1:50, sc-271540; Santa Cruz Biotechnology Inc) and anti-NCL antibody (1:50, #14,574; Cell Signaling Technology Inc). Staining with secondary antibodies, anti-rabbit IgG-Alexa488 (1:2,000, IC1051G, R&D Systems Inc, Minneapolis, MN) and anti-mouse IgG-Cy3 (1:2,000, 115–166-071, Jackson ImmunoResearch Laboratories, West Grove, PA), followed. Hoechst 33342 solution (Invitrogen Corporation) was utilized to stain the nuclei. Fluorescence was captured using the LSM 800 confocal laser scanning microscope (Carl Zeiss, Jena, Germany) at an original magnification of 63x.

### Prediction of MSLN and NCL peptides

The binding scores of 9-amino acid peptides derived from MSLN and NCL with HLA-A*02 were predicted utilizing a suite of algorithms: NetMHC [29], NetMHCpan [30], NetMHCcons [31], NetCTLpan [32], and PickPocket [33]. MSLN (MSLNp) and NCL (NCLp) peptides were selected for synthesis upon demonstrating favorable binding scores in three or more of the above algorithms. Specifically, two peptides for each protein were chosen: MSLNp number 1 (pM-01, SLLFLLFSL) and MSLNp number 2 (pM-02, VLPLTVAEV); and NCLp number 1 (pN-01, KMAPPPKEV) and NCLp number 2 (pN-02, VLSNLSYSA). These peptides were synthesized by GenScript Biotech Pte Ltd (Galaxis West Lobby, Singapore). Following synthesis, all peptides were dissolved in dimethyl sulfoxide (DMSO) to prepare a 14 mg/ml stock solution, which was then stored at -20 °C for use in subsequent experiments.

### Peptide-HLA binding simulation by molecular dynamics

The crystal three-dimensional structure of HLA-A*02 (PDB ID: 5C07), retrieved from the RCSB Protein Data Bank, served as the foundational template for molecular dynamics (MD) simulation with the synthetic peptides, MSLNp and NCLp [34]. Utilizing the Discovery Studio program, modifications were made to the structures of both HLA and the peptides to facilitate the construction of MD simulations of HLA-A*02 in a complex with either MSLNp or NCLp. Hydrogen atoms were integrated into the protein’s crystal structure of the protein via the LEaP module from the AMBER software suite. The amino acid sites within the structures of HLA-A*02 and the peptides were then parameterized using the AMBER ff14SB force field for the protein and the generalized AMBER force field (GAFF2) for the peptides.

Following the incorporation of hydrogen atoms, the complex geometries underwent energy minimization, initially through 1000 steps employing the steepest descents (SD) approaches method, followed by 3000 steps utilizing conjugated gradient (CG) methods. The molecular systems were solvated with water molecules using the TIP3P water model and neutralized by the addition of countering (Na^+^ and Cl^−^). Subsequently, the entire model system undertook a 100 ns MD simulation.

The stability of the system over the simulation period was monitored and quantified by calculating the root-mean-square deviation (RMSD) for the entirety of the 100 ns. From the conducted simulations, only snapshots extracted approximately 20 ns into the simulation were selected for the calculation of the peptide-HLA complex’s binding affinity. This was achieved through the application of the molecular mechanics energies combined with the generalized Born and surface area solvation (ΔG MMGBSA) methods to provide detailed insights into the interaction dynamics between the peptides and HLA-A*02.

### Generation of MSLN/NCL-specific T cells

PBMCs from HLA-A*02 positive HDs were utilized to generate MSLN/NCL-specific T cells and assess their immunogenicity. Initially, PBMCs were separated from peripheral blood employing a lymphocyte separation medium (Corning, NY) and subsequently cultured in 12-well plates using a density of 2 × 10^6^ cells in AIM-V medium (Thermo Fisher Scientific) supplemented with interleukin-2 (IL-2) at a concentration of 20 ng/mL, interleukin-7 (IL-7) at 10 ng/mL, interleukin-15 (IL-15) at 10 ng/mL (all from ImmunoTools GmbH, Friesoythe, Germany), and 10% human AB serum (Merck KGaA, Darmstadt, Germany). The PBMCs were stimulated with peptides at a concentration of 25 µM for 9 days, employing three cycles of stimulation [35]. On the ninth day, the activated T cells were allowed a rest period in a serum-free medium for 24 h to prepare them for subsequent further experimental analyses.

To measure T cell expansion via the TCR, monoclonal anti-CD3/anti-CD28 antibodies (anti-CD3 antibody (anti-human, pure-functional grade, clone OKT3, Miltenyi Biotec, Bergisch Gladbach, Germany) and anti-CD28 antibody (anti-human, pure-functional grade, clone 15E8, Miltenyi Biotec, Bergisch Gladbach, Germany)) were used in comparison to those of activated T cells treated with single peptide, combined peptides, and non-activated T cells or unpulsed T cells. PBMCs containing 2 × 10^6^ cells were placed into the culture medium as described above, and subsequent culture of these cells with 10 µg/ml of anti-CD3/anti-CD28 antibodies for 3 days. Cells were then stimulated by either pN-02 or pM-02 + pN-02 at a concentration of 25 µM peptide. Nine days after three cycles of restimulation, cells were detected i) CD3, CD4, CD8, CD69, and IFN-γ by flow cytometry and ii) IFN-γ production by Human IFN-γ ELISpot^BASIC^ Kit (3420-2A, MABTECH AB, Nacka, Sweden).

### T cell subset and T cell activation analysis using flow cytometry

On day 10, the activated T cells were harvested and initially blocked with 5% human AB serum (Merck KGaA). Following a washing step with 1X PBS, cells were subjected to staining with a panel of fluorochrome-conjugated antibodies. For T cell subset, panel included CD3-eFluor450 (48–0037-42, Thermo Fisher Scientific), CD4-Alexa Fluor 700 (56–0049-42, Thermo Fisher Scientific), CD8-APC-Cy7 (A15448, Thermo Fisher Scientific), CD45RA-APC (21819456, ImmunoTools GmbH), and CD62L-PE (21819624, ImmunoTools GmbH), all used at a 1:50 dilution and incubated for 30 min. For T cell activation, cells were stained with anti-CD3, anti-CD4, anti-CD8 and anti-CD69-PerCP (cMA1-10277, Thermo Fisher Scientific). Following staining, cell detection was performed utilizing flow cytometry (CytoFLEX, Beckman Coulter). Analysis of the flow cytometry data was carried out using FlowJo VX software (Treestar Inc, Ashland, OR). The results were expressed as the mean fluorescence intensity (MFI) of each specific marker, normalized by fluorescence-matched isotype control antibodies, to accurately gauge the expression levels of these markers within the population of activated T cells.

### Enzyme-linked immunosorbent spot assay

The production of IFN-γ by MSLN/NCL-specific T cells was evaluated via the enzyme-linked immunosorbent spot technique using a Human IFN-γ ELISpot^BASIC^ Kit, adhering to the manufacturer’s guidelines. In brief, MultiScreen HTS IP plates, activated with 35% ethanol and featuring a 0.45-μm Immobilon-P membrane, were coated with 15 µg/mL of anti-IFN-γ capture antibody. On the following day, 1 × 10^4^ HLA-A*02 T2 cells, having been pulsed with 25 µM of MSLNp and/or NCLp for 2 h, then, were co-incubated with 2 × 10^5^ MSLN-specific, NCL-specific, or MSLN/NCL-specific T cells. After a 2 h period, the peptide-pulsed T2 cells along with the effector T cells were transferred into the prepared ELISpot plates. Phorbol 12-myristate 13-acetate (PMA) and Ionomycin served as the positive controls, while T2 cells alone functioned as the negative control. The secretion of IFN-γ was monitored over a 24 h incubation period.

The IFN-γ spots were visualized using a biotinylated monoclonal antibody (Mab 7-B6-1) for detection, followed by the application of alkaline phosphatase (ALP)-conjugated streptavidin. For the final step, 100 µL of 0.45 µm filtered BCIP/NBT-plus solution (Mabtech AB, Nacka, Sweden) was added to the wells and left to react for 5–20 min. Following the completion of the color development reaction, the ELISpot plate was thoroughly dried in preparation for analysis. The analysis was conducted using a Bioreader 5000 Pro-F Gamma ELISPOT Reader (BioSys GmbH, Karben, Germany).

### Cancer cell killing by colony formation assay

Target cells, including MSLN^−^/NCL^+^-231, MSLN^−^/NCL^−^-231, MSLN^+^/NCL^+^-231, MSLN^+^/NCL^−^-231, and MSLN^−^/NCL^−^-10A, were assayed at a density of 1 × 10^4^ cells to evaluate their vulnerability to the cytotoxic effects of MSLN-specific, NCL-specific, or MSLN/NCL-specific T cells. These were orchestrated across different ratios of effector T cell to target cell, specifically at 1:1, 5:1, and 10:1. Following a 24 h incubation period, effector T cells were carefully removed. The residual target cancer cells were then fixed with absolute methanol and stained with a 0.5% crystal violet solution for 15 min. After staining, the cells underwent two washes with sterile deionized water to remove excess stains. The resultant colonies were imaged and quantitatively assessed using the CellCounter software (Nghia, Ho), version 0.2.1 (https://nghiaho.com/), and enabling precise evaluation of the cytotoxic capacity of the T cells against the cancer cell lines.

### Multiplex cytokine bead array

In this assay, unpulsed, MSLN-specific, NCL-specific, or MSLN/NCL-specific T cells were co-cultured with target cancer cells at an effector-to-target ratio of 10:1 for a duration of 24 h. The culture supernatants were then harvested and subjected to centrifugation for the removal of cell debris before proceeding with cytokine analysis. The cytokine concentrations within the supernatants were determined using the LEGENDplex Human CD8/NK Cell Panel (#741,065, BioLegend, CA). This multiplex assay facilitates the simultaneous quantification of 13 human cytokines and proteins, namely IL-2, IL-4, IL-6, IL-10, IL-17A, IFN-γ, TNF-α, soluble Fas, soluble FasL, granzyme A, granzyme B, perforin, and granulysin. The prepared samples were analyzed using a CytoFLEX flow cytometer (Beckman Coulter), providing a comprehensive profile of the cytokine production by the T cells in response to their interaction with cancer cell targets.

### Statistical analysis

The correlation between MSLN and NCL expressions with clinicopathological characteristics of patients were assessed using the Chi-square test and Fisher’s exact test. The prognostic significance of MSLN and NCL expressions on the survival time of patients was evaluated using the Kaplan–Meier method and log-rank test. Each study was conducted with at least three independent experiments to ensure reproducibility. All data are presented as mean ± standard deviation. Statistical analyses were carried out using IBM SPSS Statistics, version 25 (IBM Corp, Armonk, NY) and GraphPad Prism, version 9.0 (GraphPad Software Inc, San Diego, CA). A *P*-value of less than 0.05 was considered indicative of statistically significant data. For comparisons involving two samples, a Student’s t-test was applied, while for analyses involving more than two sample groups, One-way ANOVA followed by Tukey’s post-hoc test were utilized to ascertain differences.

## Results

### Co-expression of MSLN and NCL in TNBC tissues and clinicopathological correlation

An analysis of the clinicopathological characteristics of 149 TNBC patients revealed an age distribution ranging from 25 to 86 years, with a median age of 55 years. In this study, the patient cohort did not include individuals with well-differentiated histological grades of TNBC, reflective of the sampling’s random nature spanning the years between 2006 and 2021. The majority (approximately 70%) of the patients presented with poorly differentiated histological grades which corresponded to the previous study [20]. This observation aligns with findings from prior studies indicating that TNBC cases frequently exhibit poorly differentiated histological grades, with TNBC representing the subtype with the highest proportion of poorly differentiated tumors among all breast cancer subtypes [36]. Additionally, lymphovascular invasion was noted in 27.5% of patients, lymph node metastasis in 34.9%, and distant metastasis in 14.8% (Table 1).

**Table 1:** The demographical and clinicopathological data of 149 TNBC patients

Clinical characteristics	N (%)
**Age (*n* = 149)**	
≤ 50	56 (37.6%)
> 50	93 (62.4%)
**Pathological T stage (*n* = 149)**	
pT1-2	140 (94.0%)
pT3-5	9 (6.0%)
**Pathological N stage (*n* = 149)**	
pN0	98 (65.8%)
pN1-3	51 (34.2%)
**Tumor size (*n* = 149)**	
≤ 1.9 cm	39 (26.2%)
≤ 4.9 cm	100 (67.1%)
≥ 5 cm	10 (6.7%)
**Histological grade (*n* = 149)**	
Moderately differentiated	45 (30.2%)
Poorly differentiated	104 (69.8%)
**Lymphovascular invasion (*n* = 149)**	
Absence	108 (72.5%)
Presence	41 (27.5%)
**Lymph node metastasis (*n* = 149)**	
Absence	97 (65.1%)
Presence	52 (34.9%)
**Distant metastatic (*n* = 149)**	
Absence	108 (72.5%)
Presence	22 (14.8%)
No data	19 (12.7%)
**Local recurrence (*n* = 149)**	
Absence	118 (79.2%)
Presence	13 (8.7%)
No data	18 (12.1%)

**Fig. 1:**
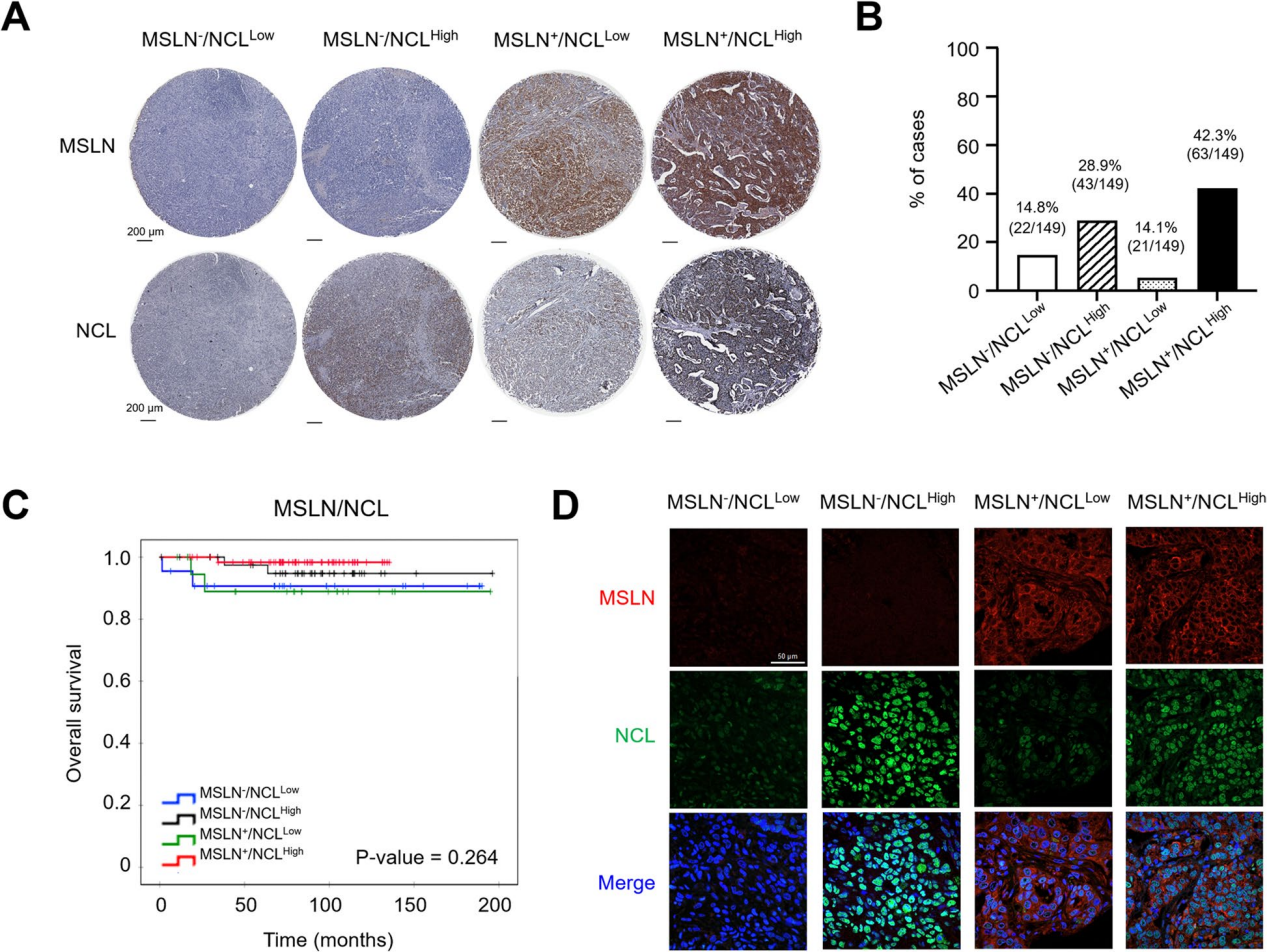
Immunohistochemical and Immunofluorescence analysis of MSLN and NCL expression in TNBC tissue samples. **a** Immunohistochemistry (IHC) demonstrates the variability of MSLN and NCL expression across TNBC tissues categorized into four groups: MSLN^−^/NCL^Low^, MSLN^−^/NCL^High^, MSLN^+^/NCL^Low^, and MSLN^+^/NCL^High^. The scale bar equals 200 µm, with original magnification at 10x. **b** Graph illustrating the percentage distribution of MSLN and NCL expression among 149 TNBC cases. **c** Kaplan–Meier survival plots contrasting the outcomes associated with different expression patterns of MSLN and NCL in TNBC patients. **d** Dual-color immunofluorescence staining identifying MSLN and NCL expressions within TNBC tissues (MSLN^−^/NCL^Low^, MSLN^−^/NCL^High^, MSLN^+^/NCL^Low^, and MSLN^+^/NCL^High^). MSLN is marked with red fluorescence, NCL with green fluorescence, and nuclei are labeled with DAPI emitting blue fluorescence. The scale bar equals 50 µm, with original magnification at 63x

The co-expression levels of MSLN and NCL in the 149 TNBC tissues [20, 28] were re-analyzed. MSLN expression was observed in the cytoplasm and the membrane of cancer cells, whereas NCL expression was identified in the cytoplasm and nucleus (Fig. 1A). Among the patients, 42.3% (63/149) exhibited co-expression of both MSLN and NCL (MSLN^+^/NCL^High^), whereas 14.8% (22/149) did not express either of the two markers (MSLN^−^/NCL^Low^). Furthermore, 28.9% (43/149) showed expression of NCL alone (MSLN^−^/NCL^High^), and 14.1% (21/149) expressed only MSLN (MSLN^+^/NCL^Low^; Fig. 1B). Kaplan–Meier survival analysis indicated that the presence of MSLN^+^/NCL^High^ cases was not significantly correlated with the overall survival time of TNBC patients (*P*-value = 0.264; Fig. 1C). Notably, co-expression of MSLN^+^ /NCL^High^ was significantly associated with histological grade (*P*-value = 0.009), the presence of lymphovascular invasion (*P*-value = 0.002), and local recurrence (*P*-value = 0.017; Table 2).

**Table 2:** The correlation between MSLN and NCL expressions in TNBC patients and the clinicopathological features

Clinical characteristics	No. of patients				*P*
	**MSLN**^**−**^**/NCL**^**Low**^	**MSLN**^**−**^**/NCL**^**High**^	**MSLN**^**+**^**/NCL**^**Low**^	**MSLN**^**+**^ **/NCL**^**High**^	
**Age (*n* = 149)**					
≤ 50 (*n* = 56)	7	16	9	24	0.903
> 50 (*n* = 93)	15	27	12	39	
**Pathological T stage (*n* = 149)**					
pT1—2 (*n* = 140)	20	40	19	61	0.618
pT3—5 (*n* = 9)	2	3	2	2	
**Pathological N stage (*n* = 149)**					
pN0 (*n* = 98)	14	30	11	43	0.533
pN1—3 (*n* = 51)	8	13	10	20	
**Tumor size (*n* = 149)**					
≤ 1.9 cm (*n* = 39)	5	14	4	16	0.792
≤ 4.9 cm (*n* = 100)	15	25	16	44	
≥ 5 cm (*n* = 10)	2	4	1	3	
**Histological grade (*n* = 149)**					
Moderately (*n* = 45)	13	8	6	18	0.009*
Poorly (*n* = 104)	9	35	15	45	
**Lymphovascular invasion (*n* = 149)**					
Absence (*n* = 108)	16	29	9	54	0.002*
Presence (*n* = 41)	6	14	12	9	
**Lymph node metastasis (*n* = 149)**					
Absence (*n* = 97)	14	30	11	42	0.573
Presence (*n* = 52)	8	13	10	21	
**Distant metastatic (*n* = 130)**					
Absence (*n* = 108)	11	31	13	53	0.126
Presence (*n* = 22)	4	8	5	5	
**Local recurrence (*n* = 131)**					
Absence (*n* = 118)	11	34	16	57	0.017*
Presence (*n* = 13)	4	6	2	1	

^*^P values less than 0.05 were considered statistically significant

To further substantiate the co-expression of MSLN and NCL in TNBC cancer cells, double immunofluorescence staining was conducted on TNBC tissues characterized as MSLN^−^/NCL^Low^, MSLN^−^/NCL^High^, MSLN^+^/NCL^Low^, and MSLN^+^/NCL^High^. The findings unambiguously demonstrated that colocalization of MSLN and NCL was prevalent in the MSLN^+^/NCL^High^ TNBC tissue specimens (Fig. 1D).

### Peptide-HLA binding prediction and production of MSLN/NCL-specific T cells

**Fig. 2:**
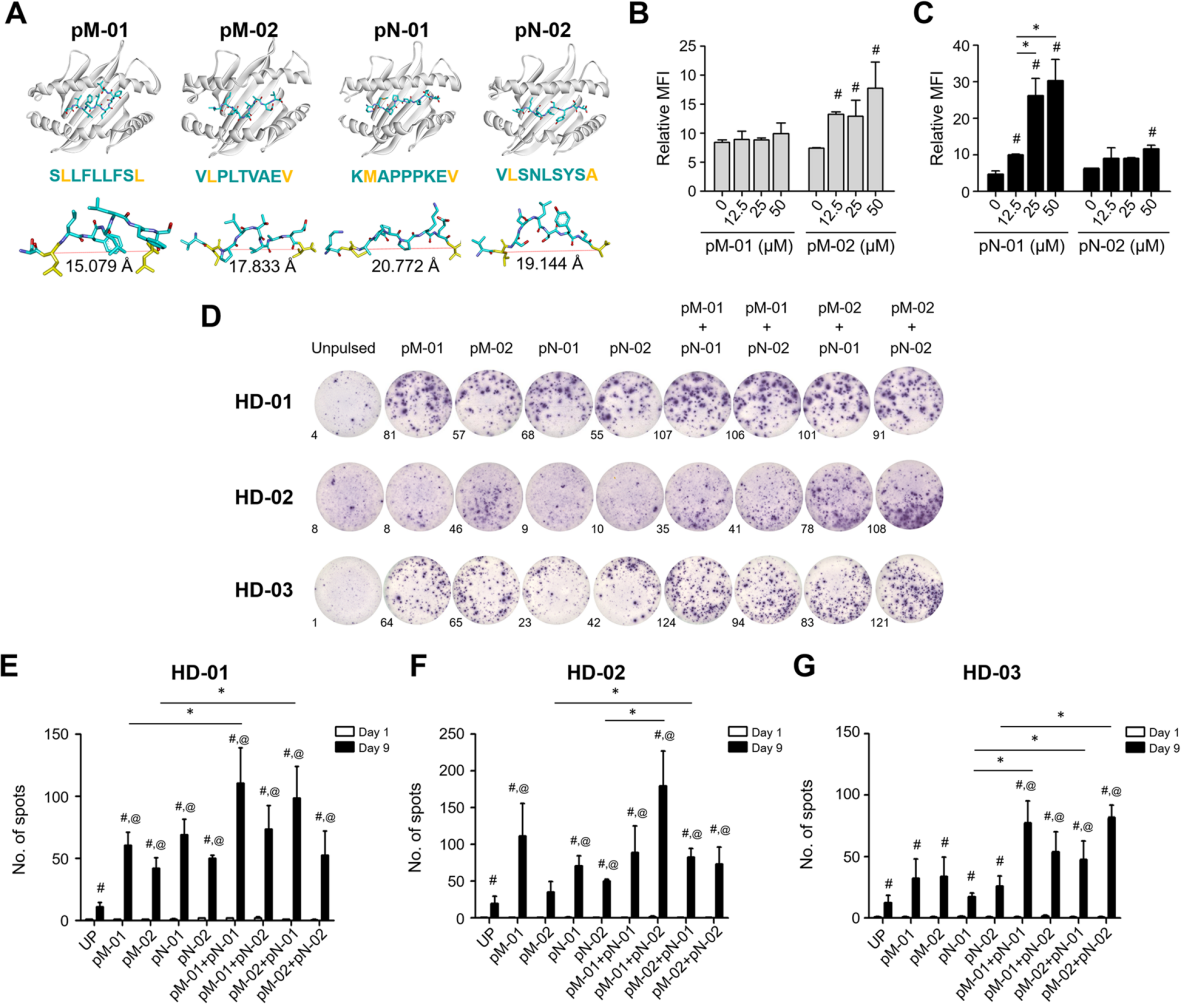
Analysis of peptide-HLA binding and generation of MSLN/NCL-specific T cells. **a** Molecular dynamics (MD) simulations illustrate the interaction between HLA-A*02 and the peptides pM-01, pM-02, pN-01, and pN-02, respectively. **b** Assessment of T2 cell binding to pM-01 and pM-02, and **c** pN-01 and pN-02 peptides at concentrations of 0, 12.5, 25, and 50 µM. **d** Evaluation of IFN-γ production by antigen-specific T cells in reaction to the respective peptides, as determined using the ELISpot assay. A representative ELISpot well image from three independent experiments is shown. **e–g** Quantitative analysis of the number of IFN-γ spots produced by T cells from healthy donors (HD-01, HD-02, and HD-03) in response to peptide stimulation. *P-value < 0.05 denotes statistical significance compared to unpulsed T cells. #*P*-value < 0.05 indicates significance compared to the control. @*P*-value < 0.05 represents statistical significance observed between day 0 and day 9. HD, healthy donor; pM-01, MSLN peptide number 1; pM-02, MSLN peptide number 2; pN-01, NCL peptide number 1; pN-02, NCL peptide number 2

Molecular dynamics (MD) simulation outcomes demonstrated the distances between the second and ninth amino acid residues, which were 15.079 angstroms (Å) for pM-01, 17.833 Å for pM-02, 20.772 Å for pN-01, and 19.144 Å for pN-02 (Fig. 2A). These distances are indicative of a suitable conformation for peptide binding to HLA-A*02 [37]. The presence of negative ΔG MMGBSA values is indicative of spontaneous binding between the HLA molecule and peptides [38]. The calculated ΔG MMGBSA values for the interactions of pM-01, pM-02, pN-01, and pN-02 with HLA-A*02 were -11.5833, -9.5812, -16.0722, and -10.0053 kcal/mol, respectively, signaling favorable binding affinities (Table 3).

**Table 3:** Characterization of MSLN and NCL peptides restricted to HLA-A*02:01

Candidates	Sequences	NetMHC	NetMHCpan	NetMHCcons	NetCTLpan	PickPocket	∆G MMGBSA (kcal/mol)	2nd-9th distance (Å)
pM01	SLLFLLFSL	0.07	0.414	0.3	0.20	0.824	-11.5833	15.079
pM02	VLPLTVAEV	0.70	0.217	1.5	0.80	0.701	-9.5812	17.833
pN01	KMAPPPKEV	1.80	0.047	4.00	1.50	0.607	-16.0722	20.772
pN02	VLSNLSYSA	0.50	0.822	1.50	1.50	0.632	-10.0053	19.144

*NetMHC* NetMHCpan, and NetMHCcons: both strong bind (%R ≤ 0.5) and weak bind (%R < 0.5) = “passed”, NetCTLpan: the threshold for binding epitope identification = 1. PickPocket: a value of 1-log50K(aff) higher than 0.5 was considered as “passed.” The underlined meet the pass criteria. Å, angstrom

The T2 cell-peptide binding test demonstrated the binding affinity of all peptides to the HLA-A*02:01 was performed. The 0, 12.5, 25, and 50 µM peptides were found to bind to HLA-A*02:01 molecules with varying affinities (Fig. 2B, C). Specifically, pM-02 had significantly high affinity binding to HLA-A*02:01 in a dose-dependent manner, with the relative MFI of 13.27 ± 0.95 at 12.5 µg/ml, 13.25 ± 3.85 at 25 µg/ml, and 18.95 ± 4.76 at 50 µg/ml, compared to those of untreated cells (Fig. 2B). The pN-01 revealed significantly high affinity binding to HLA-A*02:01 with the relative MFI of 10.73 ± 0.15 at 12.5 µg/ml, 25.13 ± 5.82 at 25 µg/ml, and 30.15 ± 5.55 at 50 µg/ml, compared to those of untreated cells (Fig. 2C). However, pM-01 and pN-02 exhibited low binding affinity to HLA-A*02:01, with relative MFI of approximately 10.90 ± 0.30 for pM-01 and 5.95 ± 2.34 for pM-01 at all concentrations (Fig. 2B, C).The administration of combined peptides demonstrated a tendency to augment IFN-γ production compared to single peptide-specific T cells across all three HDs (Fig. 2D-G). Specifically, MSLN/NCL-specific T cells activated by the combination of pM-02 with pN-01 (pM-02 + pN-01) significantly enhanced IFN-γ production compared to those treated with pM-02, or pN-01, or unpulsed T cells. The combination of pM-01 with pN-01 (pM-01 + pN-01) resulted in a significant increase in IFN-γ production compared to T cells activated by pM-01 alone in HD number 1 (HD-01; Fig. 2D, E). For HD number 2 (HD-02), MSLN/NCL-specific T cells activated with the combinations of pM-01 + pN-01, pM-01 + pN-02, pM-02 + pN-01, and pM-02 + pN-02 exhibited significantly increased IFN-γ spots compared to unpulsed T cells (Fig. 2D and F). Furthermore, in HD-02, T cells stimulated with pM-02 + pN-01 produced higher levels of IFN-γ than those stimulated with pM-02 alone; similarly, T cells activated with pM-01 + pN-02 significantly secreted more IFN-γ than those activated with pN-02 (Fig. 2F). In HD-03, T cells activated with pM-01 + pN-01 and pM-02 + pN-01 produced significantly more IFN-γ than cells exposed solely to pN-01; additionally, T cells stimulated with pM-02 + pN-02 demonstrated higher IFN-γ production than those treated with pM-02 (Fig. 2D and G).

We performed T cell expansion via the αβ-T cell receptor (TCR) with monoclonal anti-CD3/anti-CD28 antibodies [39], to compare ‘activated T cells with single peptide’, ‘activated T cells with combined peptides’, and ‘non-activated T cells or unpulsed T cells’. Briefly, the T cell numbers were measured after treatment with anti-CD3/CD28 antibodies over 9 days in pN-02 (low IFN-γ, Fig. 2D) and pM-02 + pN-02 (high IFN-γ, Fig. 2D). The results showed that these antibodies in all 3 HDs produced more IFN-γ than the same peptide treatment but with no exposure to anti-CD3/anti-CD28 antibodies (Fig. 2D; Additional File 6: Fig. S3A and S3B). There were no differences between CD8^+^ T cells and CD4^+^ T cells in response to anti-CD3/anti-CD28 antibodies (Additional File 6: Fig. S3C and S3E). Moreover, anti-CD3/anti-CD28 antibodies accumulated a significantly higher proportion of IFN-γ producing cells of both CD4^+^ and CD8^+^ T cells (Additional File 6: Fig. S3D and S3F).

### T cell subpopulation

**Fig. 3:**
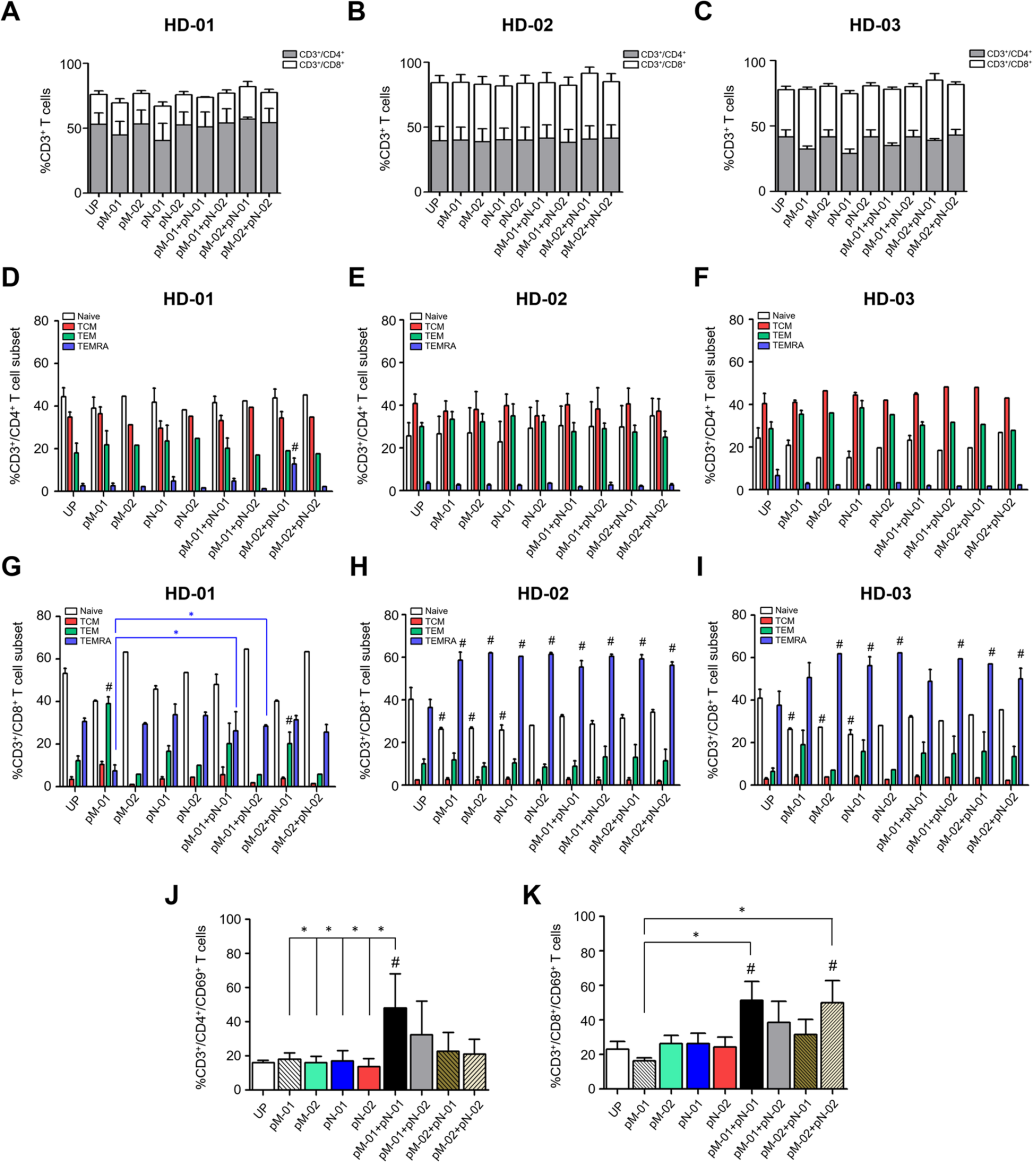
Characterization of MSLN-specific, NCL-specific, and MSLN/NCL-specific T cells in healthy donors (HDs). **a-c** Demonstrates the percentages of CD3^+^/CD4^+^ and CD3^+^/CD8^+^ T cells in healthy donors: HD-01, HD-02, and HD-03, following a specified gating strategy. **d-i** Show the distribution of naive, central memory (T_CM_), effector memory (T_EM_), and T_EMRA_ subpopulations within **d-f** CD3^+^/CD4^+^ and **g-i** CD3^+^/CD8^+^ T-cell compartments. **j** The frequencies of CD3^+^/CD4^+^/CD69^+^ and **k** of CD3^+^/CD8^+^/CD69^+^ T cell derived specific T cells after three cycle restimulation. Data were pooled from HD-01 to -03 and analyzed using one-way ANOVA and presented as mean ± SD of three independent experiments. Statistical significance is denoted by *P-value < 0.05 and #*P*-value < 0.05 when compared to unpulsed T cells. HD, healthy donor; pM-01, mesothelin peptide number 1; pM-02, mesothelin peptide number 2; pN-01, nucleolin peptide number 1; and pN-02, nucleolin peptide number 2

Only HD-01 displayed a noticeably lower quantity of CD3^+^/CD8^+^ T cells but a significantly higher quantity of CD3^+^/CD4^+^ T cells compared to HD-02 and HD-03. However, there were no significant differences in the proportions of both CD3^+^/CD4^+^ and CD3^+^/CD8^+^ T cells in PBMCs following peptide activation (Fig. 3A). Higher proportions of CD3^+^/CD8^+^ T cells relative to HD-01 were observed in both HD-02 and HD-03. Additionally, no significant differences were identified among the peptide-primed PBMCs (Fig. 3B, C).

Memory T cell subsets were analyzed following activation by treatment with either a single or a combination of MSLN and NCL peptides, and compared to the unpulsed T cells (Fig. 3D-I). Memory T cell subsets were classified as effector memory T cells (T_EM_; CD3^+^, CD8^+^, CD45RA^−^, and CD62L^−^), effector memory T cells re-expressing CD45RA (T_EMRA_; CD3^+^, CD8^+^, CD45RA^+^, and CD62L^−^), central memory T cells (T_CM_; CD3^+^, CD8^+^, CD45RA^−^, and CD62L^+^), and naive T cells (Naive; CD3^+^, CD8^+^, CD45RA^+^, and CD62L^+^). Utilizing a gating strategy, CD3^+^ T cells were divided into CD3^+^/CD4^+^ (Fig. 3D-F) and CD3^+^/CD8^+^ T cells (Fig. 3G-I). The results demonstrated significant increases in the CD3^+^/CD4^+^ T_EMRA_ population following pM-02 + pN-01 treatment, whereas pM-01 alone and pM-02 + pN-01 treatment significantly elevated the CD3^+^/CD8^+^ T_EM_ population compared to the unpulsed condition in HD-01 (Fig. 3D and G). Furthermore, the combination of both pM-01 + pN-01 and pM-01 + pN-02 resulted in a significantly enhanced proportion of CD3^+^/CD8^+^ T_EMRA_ over T cells activated with pM-01 alone in HD-01 (Fig. 3G). In HD-02, no significant differences were observed in CD3^+^/CD4^+^ and CD3^+^/CD8^+^ T cells among peptide-primed PBMCs (Fig. 3E and H). Only the proportion of CD3^+^/CD8^+^ T cells showed a decrease in naive T cells whereas T_EMRA_ increased in T cells activated with all peptide treatment conditions compared to unpulsed T cells (Fig. 3H). In HD-03, significant increases were noted in the CD3^+^/CD8^+^ T_EMRA_ population following treatment with pM-02, pN-01, pN-02, pM-01 + pN-02, pM-02 + pN-01, and pM-02 + pN-02 compared to the unpulsed condition, whereas single peptide treatments (pM-01, pN-01, and pN-02) were associated with a notable reduction in naive T cells (Fig. 3I). Nearly all peptide treatment conditions on PBMCs from HD-03 were associated with a reduction in naive T cells and an increase in the CD3^+^/CD8^+^ T_EM_ and CD3^+^/CD8^+^ T_EMRA_ populations compared to unpulsed T cells (Fig. 3I), albeit no significant changes were noted in the CD3^+^/CD4^+^ T cell population of HD-03. Notably, the lower number of CD3^+^/CD8^+^ T_CM_ and a higher number of CD3^+^/CD8^+^ T_EMRA_ compared to CD3^+^/CD4^+^ T cells was a consistent observation during the study.

### T cell activation by MSLN and NCL peptides

PBMCs were measured for the numbers of cells expressing CD3, CD4, CD8, and CD69. The percentage of CD69^+^cells was calculated by gating on CD3^+^/CD4^+^ and CD3^+^/CD8^+^ populations. The percentages were considered positive when they were at least 2 times above the background values obtained from the unpulsed controls. The frequency of CD3^+^/CD4^+^/CD69^+^ T cells was increased in all peptide combinations and that of the pM-02 + pN-02 treatment was significantly increased in comparison to those of pM-01, pN-01, and unpulsed peptide conditions (Fig. 3J). In support, the pM-01 + pN-01 activation showed a significant increase in the numbers of CD3^+^/CD8^+^/CD69^+^ T cells compared to pM-01 treated alone (Fig. 3K).

### Killing activity of the MSLN/NCL-specific T cells against 2-D TNBC cell lines

**Fig. 4:**
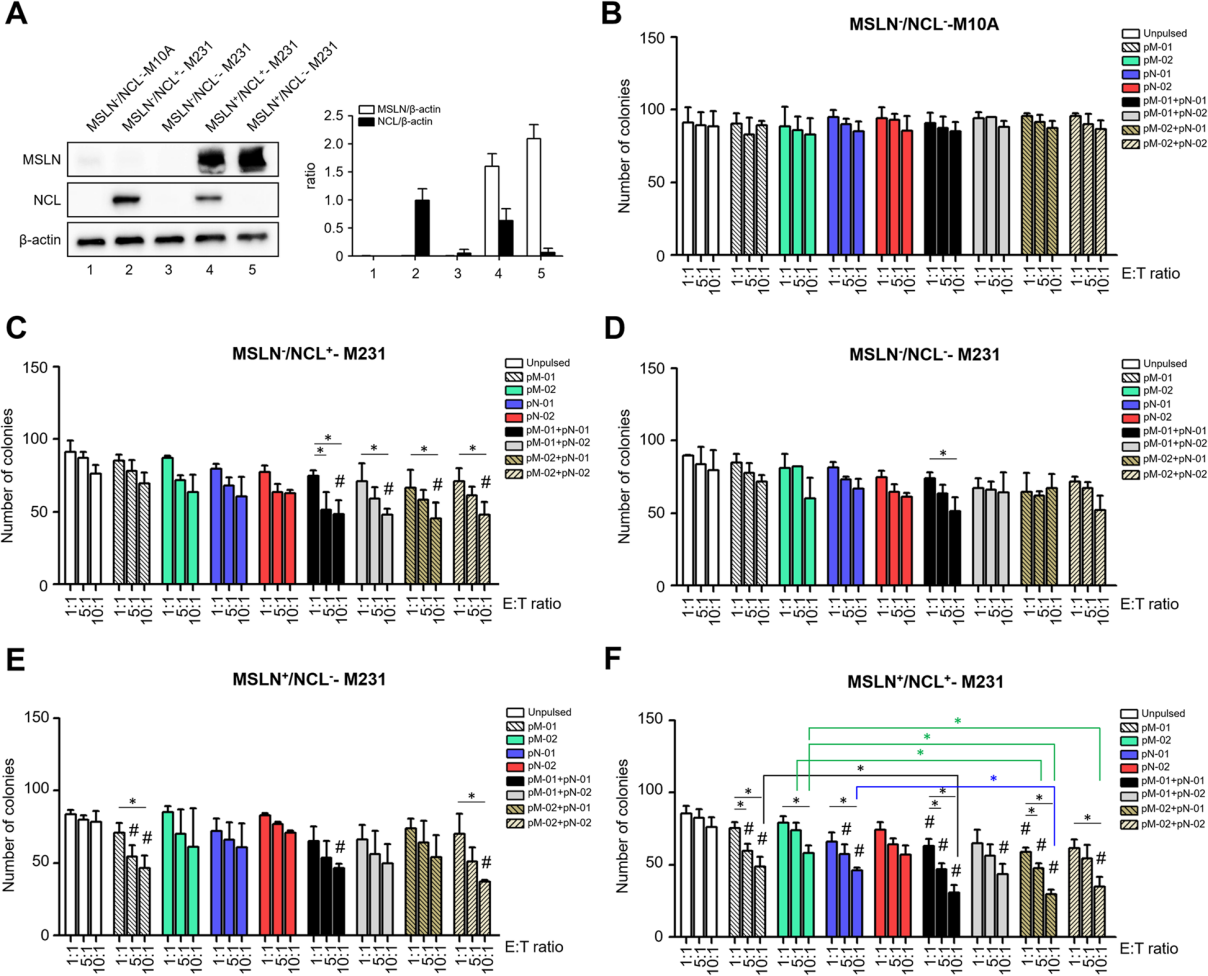
MSLN and NCL expression and the cytotoxic function of MSLN/NCL-specific T cells. **a** Presents the levels of MSLN and NCL expression in various breast cell lines: MCF-10A (MSLN^−^/NCL^−^-M10A), MDA-MB-231 (MSLN^−^/NCL^+^-M231), MDA-MB-231-NCL^KD^ (MSLN^−^/NCL^−^-M231), MSLN-overexpressing MSLN-MDA-MB-231 (MSLN^+^/NCL^+^-M231), and MSLN-MDA-MB-231-NCL^KD^ (MSLN^+^/NCL.^−^-M231), with densitometry analysis normalized against β-actin. **b-f** Depict the cytotoxic activity of MSLN/NCL-specific T cells against these five breast cell lines as determined by a colony formation assay at the indicated effector-to-target (E:T) ratios of 1:1, 5:1, and 10:1 over a duration of 24 h. The quantification of viable colonies of target cells following co-culture with T cells represents pooled results from three healthy donors (HDs). Data are derived from three independent experiments for each HD (depicted in Additional File 4: Fig. S1). Statistical significance is denoted by *P-value < 0.05 and #*P*-value < 0.05 when compared to unpulsed T cells. HD, healthy donor; KD, knockdown; pM-01, MSLN peptide number 1; pM-02, MSLN peptide number 2; pN-01, NCL peptide number 1; pN-02, NCL peptide number 2

Western blot analysis was utilized to detect MSLN and NCL levels in various MDA-MB-231 cell lines: MDA-MB-231-NCL^KD^ (MSLN^−^/NCL^−^-M231) and MSLN-MDA-MB-231-NCL^KD^ (MSLN^+^/NCL^−^-M231), along with the commercial MDA-MB-231 (MSLN^−^/NCL^+^) and MSLN-overexpressing called MSLN-MDA-MB-231 (MSLN^+^/NCL^+^) cell lines (Fig. 4A). The killing activity of T cells, activated by either single or combined peptide treatments against these five breast cancer cell lines, was assessed using a colony formation assay with crystal violet staining to determine viable cells. The representative results of crystal violet staining are depicted in Additional file 4: Fig. S1. MSLN-specific, NCL-specific, and MSLN/NCL-specific T cells exhibited no cytotoxicity against M10A normal mammary cells, which do not express MSLN or NCL (Fig. 4B). In MSLN^−^/NCL^+^-M231 cells, all peptide combinations significantly reduced the number of colonies as compared with the unpulsed group, at an effector-to-target ratio of 10:1. Additionally, all four peptide combination conditions demonstrated significant cytotoxic effects against MSLN^−^/NCL^+^-M231 cells at an effector-to-target (E:T) ratio of 10:1 compared to that E:T of 1:1 (Fig. 4C). MSLN/NCL-specific T cells from the pM-01 + pN-01 treatment showed a significantly higher cytotoxic effect at an E:T ratio of 10:1 than at 1:1 in MSLN^−^/NCL^−^-M231 cell lines (Fig. 4D). Treatment with pM-01, pM-01 + pN-01, and pM-02 + pN-02 demonstrated a significant cytotoxic effect compared to that of unpulsed T cells in MSLN + /NCL^−^-M231 cells (Fig. 4E). Furthermore, as expected, a more effective killing effect was observed in MSLN^+^/NCL^+^-M231 cells (Fig. 4F). All single and combined peptide treatments, with the exception of pM-02 and pN-02, showcased significant cytotoxic effects against MSLN^+^/NCL^+^-M231 cells compared with the unpulsed group at E:T ratios of 5:1 and 10:1. A dose-dependent killing effect was observed in all peptide-activated T cells. Moreover, comparing single and combined peptide treatments revealed that MSLN^+^/NCL^+^-M231 cells were an effective model for demonstrating a significant difference; specifically, T cells treated with pM-01 + pN-01 exhibited a higher cytotoxic effect than those treated solely with pM-01 (Fig. 4F). Similarly, treatments with pM-02 + pN-01 or pM-02 + pN-02 resulted in effector T cells exhibiting a higher cytotoxic effect against MSLN^+^/NCL^+^-M231 cells than treatments with pM-02 or pN-01 (Fig. 4F).

### Cytokine secretion from MSLN/NCL-specific T cells

**Fig. 5:**
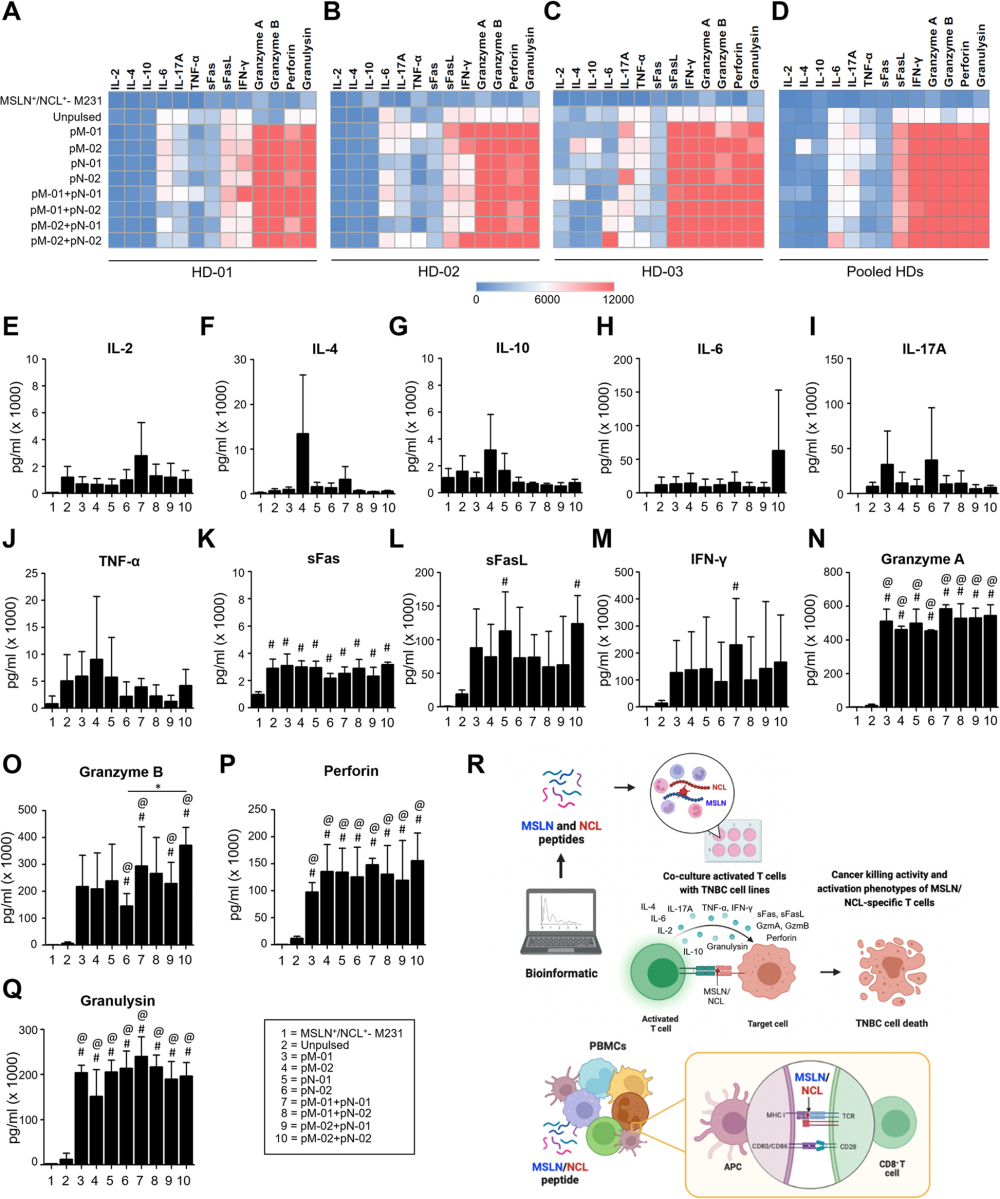
Multiplex cytokine release profile by MSLN/NCL-specific T cells. Cytokine secretion analysis via a multiplex cytokine bead array conducted following a 24 h co-culture of MSLN/NCL-specific T cells with MSLN-overexpressing MDA-MB-231 (MSLN^+^/NCL^+^-M231) cell lines at an effector-to-target (E:T) ratio of 10:1. **a-c** Display the results from an individual experiment for each healthy donor (HD) and **d** pooled data of 3 HDs, showcasing the secretion profile of a comprehensive panel of 13 cytokines, including **e** IL-2, **f** IL-4, **g** IL-10, **h** IL-6, **i** IL-17A,**j** TNF-α, **k** sFas, **l** sFASL, **m** IFN-γ, **n** granzyme A, **o** granzyme B, **p** perforin, and **q** granulysin. **r** Provides a schematic diagram elucidating the process from in silico prediction of MSLN and NCL short peptides to the presentation of these peptides by antigen-presenting cells in PBMCs via MHC molecules to T lymphocytes, culminating in a cytotoxic response. Statistical significance is denoted by #*P*-value < 0.05 when compared to target cell (MSLN^+^/NCL^+^- M231) alone; @*P*-value < 0.05 when compared to unpulsed T cells. HD, healthy donor; pM-01, MSLN peptide number 1; pM-02, MSLN peptide number 2; pN-01, NCL peptide number 1; pN-02, NCL peptide number 2

Cytokine bead array analysis was conducted to elucidate the cytokines and cytolytic molecules secreted by MSLN-specific, NCL-specific, and MSLN/NCL-specific T cells upon co-culture with MSLN^+^ /NCL^+^ -M231 cells at E:T ratio of 10:1. The analysis revealed that the levels of cytolytic molecules, including IL-2, IL-4, IL-6, IL-10, IL-17A, sFas, sFASL, TNF-α, IFN-γ, granzyme A, granzyme B, perforin, and granulysin, were significantly higher elevated in the culture medium of both the single peptide-activated T cells and the combined peptides-activated T cells compared to those of the unpulsed T cells (Fig. 5A-Q).

Significant differences were observed in the levels of granzyme A among various groups: pM-01 + pN-01 (584,217.1 ± 24,837.4 pg/ml, *P*-value < 0.001), pM-01 + pN-02 (528,316.0 ± 85,380.0 pg/ml, *P*-value < 0.001), pM-02 + pN-01 (530,615.6 ± 57,701.4 pg/ml, *P*-value < 0.001), and pM-02 + pN-02 (544,710.0 ± 64,621.0 pg/ml, *P*-value < 0.001), all demonstrating significantly higher levels than the unpulsed group (9,571.1 ± 8,558.3 pg/ml) (Fig. 5N). The levels of granzyme B were also significantly elevated in pM-01 + pN-01 (285,781.0 ± 128,958.0 pg/ml, *P*-value = 0.0108), pM-01 + pN-02 (266,041.0 ± 133,699.0 pg/ml), pM-02 + pN-01 (228,908.0 ± 78,926.0 pg/ml, *P*-value = 0.0360), and pM-02 + pN-02 (351,029.0 ± 86,484.0 pg/ml, *P*-value = 0.0204) compared to the unpulsed condition (6,261.2 ± 4,604.2 pg/ml) (Fig. 5O). The mixed pM-02 + pN-02 induced significant granzyme B secretion compared to pM-02 alone (*P*-value = 0.0468) (Fig. 5O). Perforin levels in pM-01 + pN-01 (133,812.0 ± 17,372.0 pg/ml, *P*-value = 0.0028), pM-01 + pN-02 (130,757.0 ± 52,839.0 pg/ml, *P*-value = 0.0266), pM-02 + pN-01 (119,738.0 ± 73,319.0 pg/ml, *P*-value = 0.0278), and pM-02 + pN-02 (155,740.0 ± 51,269.0 pg/ml, *P*-value = 0.0400) were significantly higher than those in the unpulsed group (11,900.0 ± 3,691.6 pg/ml) (Fig. 5P). Similarly, granulysin levels were significantly elevated in pM-01 + pN-01 (240,365.5 ± 43,156.5 pg/ml, *P*-value < 0.001), pM-01 + pN-02 (203,715.0 ± 45,407.0 pg/ml, *P*-value < 0.001), pM-02 + pN-01 (190,088.8 ± 38,702.8 pg/ml, *P*-value < 0.001), and pM-02 + pN-02 (196,692.0 ± 29,633.0 pg/ml, *P*-value < 0.001) compared to the unpulsed group (12,269.0 ± 13,364.0 pg/ml) (Fig. 5Q). The cytokine and cytolytic molecule release profiles of all peptide-specific T cells during co-culture with MSLN^+^/NCL^+^-M231 cells are summarized in Additional file 3: Table S3.

We described the process as schematic diagram elucidating in silico prediction of MSLN and NCL short peptides to the presentation of these peptides by antigen-presenting cells via MHC molecules to T lymphocytes, culminating in a cytotoxic responses (Fig. 5R).

## Discussion

Triple-negative breast cancer (TNBC), representing approximately 15%–20% of all breast cancers, is characterized by a dismal prognosis, marked by its aggressive phenotype with considerable metastatic potential and a high risk of recurrence. This subtype exhibits significant intra-tumoral heterogeneity, further complicating its management [2, 3, 40, 41]. TNBC lacks expression of hormonal receptors, specifically the estrogen receptor, progesterone receptor, and human epidermal growth factor receptor 2 (HER2), rendering it insusceptible to targeted endocrine or anti-HER2 therapies [41]. Chemotherapy has traditionally served as the cornerstone of treatment for patients with TNBC; however, only about one-third of these patients exhibit responsiveness to such treatment. Moreover, chemotherapy may lead to suboptimal long-term outcomes due to the considerable likelihood of disease recurrence [42]. The high immunogenicity of TNBC, compared to other molecular subtypes, is attributed to its elevated tumor mutational burden and the presence of tumor-infiltrating immune cells. Consequently, cancer immunotherapy, which enhances the patient’s immune response against the tumor, has emerged as a vital alternative in the treatment paradigm for TNBC [5].

Adoptive cell immunotherapy, leveraging ex vivo expanded antigen-specific T cells, is a highly promising cancer treatment modality due to its specificity, self-replicating nature, and potential lack of toxicity [9]. Synthetic peptides serve as an accessible and convenient means for stimulating T cells. One effective technique involves stimulating peripheral blood mononuclear cells (PBMCs) with synthetic peptides to establish antigen-specific T cells [43, 44]. This method offers several advantages, including ease of synthesis, no specific accumulation in organs, and minimal toxic side effects [45]. Nonetheless, the targeting of a single antigen by antigen-specific T cells may be insufficient to eliminate all TNBC cells, attributed to the tumor’s extensive heterogeneity [13–15]. Peptide-pulsed PBMC-based immunotherapy has become one of the hot topics in research targeting therapies for solid tumors, partly due to the comprehensive array of intracellular mechanisms of cross-presentation by antigen-presenting cells within PBMCs, including dendritic cells (DCs), B cells, monocytes/macrophages, and platelets, alongside its practicability [46]. To date, in vitro stimulation of PBMCs with combined peptides has successfully generated multi-peptide-specific T cells. These cells display cytotoxic activity against both hematopoietic malignancies [13–15] and solid tumors, including breast cancer [16]. The simultaneous targeting of multiple antigens has been shown to be safe, to induce disease stabilization, and to potentially diminish the risk of tumor immune evasion. Such outcomes underscore the extended value of adoptive T cell therapy in the clinical setting [13–16]. Furthermore, we envision that peptide-pulsed PBMC-based adoptive cell therapy has a bright future. The generation of T cell priming with exogenous antigen-specific T cells from PBMCs presents a lot of advantages including the increased central memory CD8 + T cells (TCM) [47], in comparison to very few CD8^+^ TCM found in the adoptive transfer of tumor-infiltrating lymphocytes (TILs) [48]. Moreover, they have more capacity as cytolytic effector cells in the multi-peptides-based method compared to the report of no-to-little perforin and granulysin generated from TILs [49].

Several overexpressed antigens on TNBC cells can be recognized by T cells, offering potential targets for cancer immunotherapy [23]. Among these mesothelin (MSLN) and nucleolin (NCL) have been identified as promising targets due to their significant overexpression of around 34–67% for MSLN and 75%–80% for NCL in clinical samples of TNBC [20, 23]. As a result, MSLN and NCL represent viable candidates for T cell-specific approaches in treating TNBC patients. In the current study, a high incidence of MSLN and NCL positivity (85.3%) was detected among TNBC patients. In terms of clinicopathological correlation, our group has previously demonstrated that elevated levels of NCL in TNBC samples were associated with shorter survival times for patients [20], whereas no significant association was found between MSLN expression and the overall survival of TNBC patients [28]. Our earlier analyses also revealed that high levels of both MSLN and NCL were associated with poor differentiation in TNBC.

Crucially, beyond the prognostic implications of co-expressed MSLN and NCL, a high proportion of TNBC patients have been found to co-express both MSLN and NCL. These findings support the rationale for simultaneously targeting MSLN and NCL using multi-peptide-specific T cells in the treatment of TNBC. The generation of MSLN/NCL-specific T cells involved the simultaneous pulsing of PBMCs with short peptides of MSLN and NCL, which were selected based on their highest binding scores with HLA-A*02:01 in MDA-MB-231 [50]. This selection was informed by bioinformatic algorithms, including NetMHC, NetMHCpan, NetMHCcons, NetCTLpan, and PickPocket [31, 51–53], and confirmed through molecular dynamics simulations of peptide binding with HLA-A*02 [54, 55]. The epitopes of MSLN peptides, specifically tailored for cytotoxic T lymphocytes (CTLs) clones, shared sequences previously demonstrated to exert an anti-tumor effect against MSLN-expressing malignant pleural mesothelioma [56] and pancreatic cancer cells [57]. In the present investigation, the selected MSLN and NCL peptides exhibited spontaneous and strong binding to HLA-A*02 molecules, indicating their appropriateness as candidates for T cell stimulation. In the context of tumor antigen peptides, the cross-reactivity could be potentially found in T cells generated against peptides and bind to other proteins or peptides in the body that share similar epitopes resulting in an autoimmune response [58, 59]. In the present study, our candidate MSLN and NCL peptides could recognize and activate T cells in the peptide-HLA complex presented on APCs, which could lead to the generation of T cells specific to MSLN and NCL epitopes. We found the evidence supported that no indications of autoimmune reaction were found in any of the 15 patients after the administration of Phase I study of MSLN-targeting T cells [60]. Furthermore, the distances between the second and ninth amino acid residues, ranging from 15–21 Å, have been supported by evidence as predictors of strong binding capability to HLA class I molecules [37]. The stability of MHC-peptide complexes can be affected by pH changes. Implied by its effect on stability and binding of MHC-II molecules [61]. However, in this study, the results from flow cytometry of T2-peptide binding assay and kinetics and affinity of peptide binding to MHC class I molecules in real-time by MD had provided the information that our candidate peptide have strong binding to HLA complexes during the various washing buffers and pH changed condition in the culture system.

Collectively, the in silico analyses underscore that pM-01, pM-02, pN-01, and pN-02 serve as suitable candidate peptides for stimulating PBMCs, potentially leading to the production of MSLN/NCL-specific T cells. We carefully designed two MSLN and two NCL candidate short peptides in silico and ensured the MHC-I (HLA-A*02) binding by the positive anti-tumor T cell response. To clarify their MHC-II binding, we carefully checked and confirmed that four short peptides could not bind with MHC-II by NetMHCII tool (https://services.healthtech.dtu.dk/services/ NetMHCII-2.3/). PBMCs from three HLA-A*02 HDs were stimulated with combined MSLN and NCL peptides for 9 days through three stimulation cycles, which resulted in an enhanced production of IFN-γ compared to both single peptide-activated T cells and untreated T cells. It is established that IFN-γ can be secreted by both CD8^+^ and CD4^+^ T cells [62, 63]. Typically, in healthy individuals, the CD4^+^/CD8^+^ ratio fluctuates between 1.5:1 to 2.5:1, where an inverted ratio signifies intense chronic immune responses [64]. However, after three cycles of peptide restimulation, HDs-02 and -03 exhibited a prevalence of CD3^+^/CD8^+^ over CD3^+^/CD4^+^ T cells, while higher levels of CD3^+^/CD4^+^ T cells were only observed in HD-01. The combination peptides (pM-01 + pN-01, pM-01 + pN-02, pM-02 + pN-01, or pM-02 + pN-02) induced greater numbers of CD3^+^/CD8^+^/IFN-γ^+^ T cells than either single peptide treatment or the unpulsed control across all three HDs. These outcomes align with previous findings reported in mouse models of breast cancer [65], underscoring the potential for combined peptide treatments to significantly enhance the immunogenic response, particularly in the context of TNBC treatment.

Furthermore, CD3^+^/CD8^+^ T cells exhibited terminal effector memory T cells demonstrated a T_EMRA_ phenotype, which is immunosenescence characterized by reduced proliferation potential yet exhibiting robust cytotoxicity and proinflammatory activity [66]. Notably, CD3^+^/CD8^+^ T_EMRA_ cells are capable of producing effective effector molecules, including perforins, granzymes, IFN-γ, and TNF-α [67, 68]. Conversely, the population of memory T cells, including central memory cells (encompassing T_CM_ and effector memory T_EM_) correlated with cells that have the ability to produce IL-2 and effector cytokines upon stimulation [69], was predominantly found in the CD3^+^/CD4^+^ T cells of all three HDs. The requirement for CD4 help in initiating and sustaining a CD8 response is well recognized, leading to the rationale for generating both CD4^+^ and CD8^+^ T cell immunity using peptide-activated whole PBMCs [44, 70, 71]. Additionally, the stimulation of PBMCs with multiple peptides has highlighted the potential to generate T_CM_ and T_EM_ anti-cancer T cells. Of note, MSLN and NCL peptides-induced the expression of CD69, known as an early T cell activation marker [72], was equally high in samples obtained from 9 days after three cycles of restimulation, suggesting that CD69 expression may be possibly used for both short- (< 4 days) and long-term (≥ 4 days) monitoring of the peptide-specific helper and cytotoxicity T cells [73]. This approach is consistent with the use of multi-peptides derived from prevalently expressed antigens in conditions such as multiple myeloma [74], reflecting the utility and importance of multi-faceted immunogenic stimulation in eliciting a comprehensive anti-cancer T cell response.

Importantly, the MSLN/NCL-specific T cells did not exhibit cytotoxic effects on normal breast mammary cells and TNBC cells that were negative for both MSLN and NCL expression. Intriguingly, these MSLN/NCL-specific T cells demonstrated the highest cytotoxic activities against TNBC cells expressing both MSLN and high levels of NCL, displaying a dose-dependent efficacy. The killing activity of MSLN/NCL-specific T cells against MSLN-positive TNBC cells was significantly more efficient than that of T cells specific to MSLN or NCL alone. This observation is noteworthy and agrees with previous work that reports the efficacy of simultaneously targeting multiple tumor antigens. This strategy could potentially mitigate the challenges posed by the heterogeneous nature of tumor antigens, thereby reducing the risk of tumor immune evasion [14, 75].

Furthermore, our investigation found that MSLN/NCL-specific T cells released significantly higher levels of granzyme A, granzyme B, perforin, and granulysin following stimulation with a combination of MSLN and NCL peptides, particularly with the combinations pM-01 + pN-01 and pM-02 + pN-02, in comparison to the unpulsed PBMCs. The role of these cytokines in killing target cancer cells is well-documented [76, 77], and their release from peptide-activated T cells has been reported previously [74]. However, it is worth noting that the use of pooled peptides to pulse PBMCs, thereby driving specific T cell responses, was not examined in the present study. Stimulating HD PBMCs with such pooled peptides could potentially significantly enhance IFN-γ production, development of CD8^+^ T_EM_ and T_EMRA_ cells, and killing activity [78]. The employment of large panels of overlapping peptide pools—which consist of numerous peptide fragments of identical length and overlapping sequences—can considerably influence the outcome of T cell responses, depending on the concentration, number of peptides per pool, length of the peptides, and range of overlap [78–80]. Nevertheless, it has been reported that increasing the number of peptides per pool can impair antigen-specific CD8^+^ T cell proliferation [81], indicating a nuanced balance must be achieved to optimize T cell responses.

Though the relatively small cohort of TNBC patients analyzed in this study, the significant correlation between MSLN and NCL co-expression and patient survival was not detected. However, we found a statistically significant correlation between co-expression of MSLN^+^ and NCL^High^ was significantly associated with histological grade (*P*-value = 0.009), the presence of lymphovascular invasion (*P*-value = 0.002), and local recurrence (*P*-value = 0.017; Table 2), which reflected in poor prognosis. Moreover, molecular dynamics (MD) simulation presented by ΔG MMGBSA values showed the binding of certain MSLN/NCL peptides with MHC-I. High IFN-γ produced from T cells derived from MSLN/NCL peptides pulsing PBMCs and effective killing of these MSLN/NCL-specific T cells against MSLN/NCL-positive cancer cells imply that the overexpression of MSLN and NCL could induce MSLC/NCL-specific T cells. In support, our previous reports showed the presenting of these two proteins by DCs activated MSLN- or NCL-specific T cells [20, 28].

## Conclusions

In conclusion, the novelty of the current study revolves around the identification of four HLA-A*02-restricted epitopes of MSLN and NCL with high binding affinity and complex stability, which rendered them as potential antigenic peptides for the production of anti-cancer T cells (Additional File 5: Fig. S2). The stimulation of PBMCs with combined MSLN and NCL peptides was found to enhance the release of IFN-γ from MSLN/NCL-specific T cells, exceeding the release from single peptide-activated T cells. Furthermore, the combined peptides successfully engaged effector memory CD4^+^ and CD8^+^ T cells, which demonstrated efficient cytotoxicity against TNBC cells overexpressing MSLN and NCL in both a dose- and antigen-dependent manner. These findings suggest the potential impact of combined or multi-peptide-specific T cells for the development of adoptive T cell therapies targeting TNBC patients.

## Supplementary Information

Additional file 1: Table S1. HLA of breast cancer cells and healthy donors.

Additional file 2: Table S2. Cell lines used in this study and their MSLN and NCL levels.

Additional file 3: Table S3. The cytokine and cytolytic molecule releasing profiles.

Additional file 4: Figure S1. Evaluation of cytotoxic effects on breast cell lines via colony formation assay. The assay was conducted on five breast cell lines following a 24-h co-treatment with MSLN-specific, NCL-specific, and MSLN/ NCL-specific T cells. The breast cell lines included MCF-10A (MSLN^−^/NCL^−^-M10A), MDA-MB-231 (MSLN^−^/NCL^+^-M231), MDA-MB-231-NCLKD (MSLN^−^/NCL^−^-M231), MSLN-overexpressing MSLN-MDA-MB-231 (MSLN^+^/NCL^+^-M231), and MSLN-MDA-MB-231-NCL^KD^ (MSLN^+^/NCL^−^-M231). HD, healthy donor; pM-01, MSLN peptide number 1; pM-02, MSLN peptide number 2; pN-01, NCL peptide number 1; pN-02, NCL peptide number 2.

Additional file 5: Figure S2. Conceptual framework of the study. Created with BioRender.com.

Additional file 6: Figure S3. Improves antigen-specific T cell expansion in PBMC cultures in the presence of anti-CD3/anti-CD28 antibodies. a Evaluation of IFN-γ production by antigen-specific T cells in reaction to the respective peptides, as determined using the ELISpot assay. A representative ELISpot well image from independent experiments of each healthy donor (HD) is shown. b Quantitative analysis of the number of IFN-γ spots produced by T cells from HD-01, HD-02, and HD-03 in response to peptide stimulation after anti-CD3/anti-CD28 antibodies culture. c-d PBMCs were determined by measuring CD3/CD4/CD8/IFN-γ positive T cells in the presence of 10 µg/ml of anti-CD3/anti-CD28 antibodies by flow cytometry. e–f Demonstrates the percentages of CD3^+^/CD4^+^, CD3^+^/CD8^+^, CD3^+^/CD4^+^/IFN-γ^+^, and CD3^+^/CD8^+^/IFN-γ^+^ T cells from HD-01, HD-02, and HD-03, following a specified gating strategy.

Additional file 7. Uncropped membrane pictures.

## Data Availability

The data that support the fndings of this study are available from the corresponding author upon reasonable request.

## Abbreviations

HD: Healthy donor
HLA: Human leukocyte antigen
IFN-γ: Interferon-gamma
IL: Interleukin
MSLN: Mesothelin
NCL: Nucleolin
PBMCs: Peripheral blood mononuclear cells
PD-L1: Programmed death-ligand 1
TNBC: Triple-negative breast cancer

## Data availability

No datasets were generated or analysed during the current study.
