# Supplementary material for: Mesothelin- and nucleolin-specific T cells from combined short peptides effectively kill triple-negative breast cancer cells
Source: BMC Med. 2024 Sep 18;22:400. doi: 10.1186/s12916-024-03625-3 (PMC11411782; doi:10.1186/s12916-024-03625-3)
Supplement: Supplementary file 1 — Additional file 1: Table S1. HLA of breast cancer cells and healthy donors. [file 12916_2024_3625_MOESM1_ESM.docx]

**Table S1**. HLA of breast cancer cells and healthy donors.

| **Subjects** | **HLA** | | | | | |
| --- | --- | --- | --- | --- | --- | --- |
|  | **A*** | **A*** | **B*** | **B*** | **C*** | **C*** |
| MCF-10A | 01:01 | 33:01 | 55:01 | 40:01 | 07:02 | 03:03 |
| MDA-MB-231 | **02:17** | **02:17** | 41:01 | 40:02 | 02:02 | 17:01 |
| HD-01 | **02:01** | 24:02 | 07:05/06 | 15:02 | 07:02 | 08:01 |
| HD-02 | **02:03** | 11:01 | 15:02 | 35:01/29 | 04:01 | 08:01 |
| HD-03 | **02:07** | 11:01 | 18:01 | 54:01 | 01:02 | 07:04 |

HD, healthy donor.
