# Supplementary material for: Mesothelin- and nucleolin-specific T cells from combined short peptides effectively kill triple-negative breast cancer cells
Source: BMC Med. 2024 Sep 18;22:400. doi: 10.1186/s12916-024-03625-3 (PMC11411782; doi:10.1186/s12916-024-03625-3)
Supplement: Supplementary file 2 — Additional file 2: Table S2. Cell lines used in this study and their MSLN and NCL levels. [file 12916_2024_3625_MOESM2_ESM.docx]

**Table S2**. Cell lines used in this study and their MSLN and NCL levels.

| **Cell lines** | **MSLN status** | **NCL status** | **Name in this study** | **Source** |
| --- | --- | --- | --- | --- |
| MDA-MB-231 | Negative | Positive | MSLN^-^/NCL^+^-M231 | Commercially available |
| MDA-MB-231-NCL^KD^ | Negative | Negative | MSLN^-^/NCL^-^-M231 | Produced in this study |
| MSLN-MDA-MB-231 | Positive | Positive | MSLN^+^/NCL^+^-M231 | [28] |
| MSLN-MDA-MB-231-NCL^KD^ | Positive | Negative | MSLN^+^/NCL^-^-M231 | Produced in this study |
| MCF-10A | Negative | Negative | MSLN^-^/NCL^-^-M10A | Commercially available |
