# Supplementary material for: Mesothelin- and nucleolin-specific T cells from combined short peptides effectively kill triple-negative breast cancer cells
Source: BMC Med. 2024 Sep 18;22:400. doi: 10.1186/s12916-024-03625-3 (PMC11411782; doi:10.1186/s12916-024-03625-3)
Supplement: Supplementary file 3 — Additional file 3: Table S3. The cytokine and cytolytic molecule releasing profiles. [file 12916_2024_3625_MOESM3_ESM.docx]

**Table S3.** The cytokine and cytolytic molecule releasing profiles.

| **Samples** | **IL-2** | **IL-4** | **IL-10** | **IL-6** | **IL-17A** | **TNF-α** | **sFas** | **sFasL** | **IFN-γ** | **Granzyme A** | **Granzyme B** | **Perforin** | **Granulysin** |
| --- | --- | --- | --- | --- | --- | --- | --- | --- | --- | --- | --- | --- | --- |
| HD-01 MSLN^+^/NCL^+^-M231 | 16.6 | 16.5 | 399.8 | 268.8 | 134.9 | 1.0 | 1200.9 | 850.9 | 1140.7 | 2360.6 | 931.0 | 768.2 | 2710.7 |
| HD-01 Unpulsed | 548.9 | 322.0 | 607.1 | 7620.9 | 7620.9 | 3512.7 | 3391.5 | 23250.5 | 6046.4 | 2065.5 | 1315.0 | 11152.0 | 5915.8 |
| HD-01 pM-01 | 24.6 | 417.9 | 518.9 | 17163.8 | 3994.4 | 777.9 | 2214.1 | 29569.5 | 18632.5 | 562727.2 | 149106.8 | 81220.1 | 209085.2 |
| HD-01 pM-02 | 258.5 | 355.5 | 417.2 | 30118.2 | 3787.6 | 1973.0 | 2496.5 | 44717.4 | 41100.8 | 543707.4 | 280269.3 | 110268.0 | 247033.2 |
| HD-01 pN-01 | 197.4 | 429.4 | 273.3 | 22289.6 | 4709.0 | 2443.1 | 2422.9 | 36252.4 | 76659.0 | 556073.0 | 272067.6 | 112840.9 | 234891.0 |
| HD-01 pN-02 | 142.3 | 223.3 | 359.8 | 18051.1 | 3193.5 | 1062.6 | 2041.1 | 32062.6 | 27600.3 | 551871.4 | 189166.69 | 92173.16 | 240857.7 |
| HD-01 pM-01+pN-01 | 208.5 | 431.2 | 883.8 | 14365.3 | 7201.2 | 4976.0 | 2960.5 | 62497.4 | 147607.0 | 593102.5 | 411082.12 | 134911.1 | 252118.7 |
| HD-01 pM-01+pN-02 | 487.0 | 291.7 | 435.7 | 2501.7 | 3292.3 | 672.5 | 3322.5 | 40052.2 | 13792.1 | 608294.0 | 418872.9 | 122950.2 | 210327.3 |
| HD-01 pM-02+pN-01 | 318.2 | 331.7 | 237.6 | 6146.2 | 3304.6 | 859.4 | 1563.0 | 15191.4 | 18152.1 | 562142.8 | 151397.1 | 68417.4 | 224874.5 |
| HD-01 pM-02+pN-02 | 268.3 | 407.0 | 433.6 | 3994.0 | 4136.8 | 733.4 | 2998.2 | 35074.2 | 15549.8 | 586655.5 | 406966.0 | 109572. | 196688.0 |
| HD-02 MSLN+/NCL+-M231 | 75.6 | 463.8 | 2477.8 | 521.5 | 146.9 | 2477.8 | 911.2 | 1069.2 | 1204.0 | 2477.8 | 1016.3 | 737.2 | 2319.9 |
| HD-02 Unpulsed | 234.6 | 197.2 | 348.1 | 25154.0 | 3968.5 | 1182.1 | 2111.9 | 21538.9 | 25168.7 | 7756.5 | 10422.5 | 8638.9 | 27624.6 |
| HD-02 pM-01 | 358.8 | 561.6 | 832.4 | 21704.5 | 19166.1 | 9315.2 | 3914.4 | 89438.1 | 238161.7 | 593147.1 | 413145.3 | 158278.6 | 217637.8 |
| HD-02 pM-02 | 259.3 | 445.1 | 622.7 | 12830.6 | 5525.4 | 2650.8 | 3161.3 | 47684.1 | 102019.4 | 583833.3 | 352227.8 | 153542.6 | 198624.4 |
| HD-02 pN-01 | 145.1 | 642.1 | 468.3 | 2874.3 | 3295.8 | 557.9 | 3344.1 | 30690.8 | 13551.4 | 540046.8 | 355365.2 | 104798.4 | 168150.1 |
| HD-02 pN-02 | 331.4 | 512.6 | 390.8 | 15872.2 | 3709.5 | 330.6 | 1937.1 | 27552.9 | 16607.2 | 565684.9 | 150098.5 | 94910.0 | 230223.9 |
| HD-02 pM-01+pN-01 | 438.0 | 525.1 | 540.5 | 31634.1 | 3802.8 | 2132.4 | 2598.9 | 48054.6 | 44774.5 | 556159.1 | 413145.3 | 135588.7 | 248316.6 |
| HD-02 pM-01+pN-02 | 359.8 | 666.6 | 491.7 | 8305.0 | 3988.0 | 1437.9 | 2163.1 | 18692.6 | 24582.5 | 538249.4 | 170721.9 | 82256.0 | 245453.3 |
| HD-02 pM-02+pN-01 | 62.8 | 514.9 | 290.0 | 1797.0 | 2669.4 | 294.9 | 2769.4 | 26519.6 | 8914.3 | 452675.9 | 309176.7 | 87086.8 | 148398.3 |
| HD-02 pM-02+pN-02 | 452.6 | 507.3 | 591.4 | 18891.1 | 7046.1 | 5907.7 | 3265.7 | 66729.3 | 164812.2 | 577182.2 | 394702.8 | 146731.5 | 226326.8 |
| HD-03 MSLN+/NCL+-M231 | 65.1 | 222.8 | 522.3 | 70.4 | 1.0 | 1.0 | 857.0 | 394.6 | 939.4 | 2524.2 | 662.0 | 99.9 | 2478.4 |
| HD-03 Unpulsed | 2784.9 | 1539.1 | 3889.2 | 3192.0 | 12645.6 | 10562.1 | 3192.0 | 12507.3 | 11591.4 | 18891.1 | 7046.1 | 15907.7 | 3265.7 |
| HD-03 pM-01 | 1760.5 | 2004.3 | 1930.4 | 1776.1 | 74242.8 | 7740.3 | 3187.4 | 144523.4 | 348669.7 | 478182.8 | 54755.1 | 95611.6 | 185724.1 |
| HD-03 pM-02 | 1495.6 | 39617.7 | 8494.4 | 1275.6 | 25317.8 | 22507.8 | 3350.5 | 130607.5 | 294081.5 | 458414.9 | 236953.6 | 193627.6 | 89099.3 |
| HD-03 pN-01 | 1503.2 | 3618.4 | 4214.4 | 2603.7 | 17041.6 | 14203.8 | 3092.0 | 184661.5 | 357004.8 | 407570.3 | 88165.8 | 185630.6 | 182729.6 |
| HD-03 pN-02 | 2535.2 | 3413.9 | 1558.7 | 1884.2 | 104383.3 | 5261.6 | 2565.5 | 159551.2 | 421659.7 | 461175.8 | 95581.2 | 189436.9 | 170087.1 |
| HD-03 pM-01+pN-01 | 7760.1 | 8859.6 | 639.3 | 1466.5 | 20991.5 | 4669.3 | 2022.9 | 111734.0 | 262024.8 | 403389.8 | 40953.6 | 130928.6 | 170661.3 |
| HD-03 pM-01+pN-02 | 3038.2 | 1006.0 | 814.2 | 15947.0 | 27139.8 | 4597.2 | 3232.4 | 119766.7 | 283448.8 | 438403.6 | 208529.3 | 187064.7 | 195363.8 |
| HD-03 pM-02+pN-01 | 3241.8 | 590.7 | 999.1 | 16481.4 | 10363.2 | 2533.3 | 2643.5 | 145563.1 | 424860.1 | 464019.1 | 226150.9 | 203710.8 | 156993.6 |
| HD-03 pM-02+pN-02 | 2350.5 | 948.9 | 1244.3 | 166456.7 | 8877.1 | 5984.6 | 3305.3 | 174808.2 | 341648.5 | 470293.6 | 251416.6 | 210917.1 | 167060.3 |

HD, healthy donor; pM-01, MSLN peptide number 1; pM-02, MSLN peptide number 2; pN-01, NCL peptide number 1; pN-02, NCL peptide number 2.Unit was pg/ml.
