# Supplementary figures and images for: Mesothelin- and nucleolin-specific T cells from combined short peptides effectively kill triple-negative breast cancer cells
Source: BMC Med. 2024 Sep 18;22:400. doi: 10.1186/s12916-024-03625-3 (PMC11411782; doi:10.1186/s12916-024-03625-3)

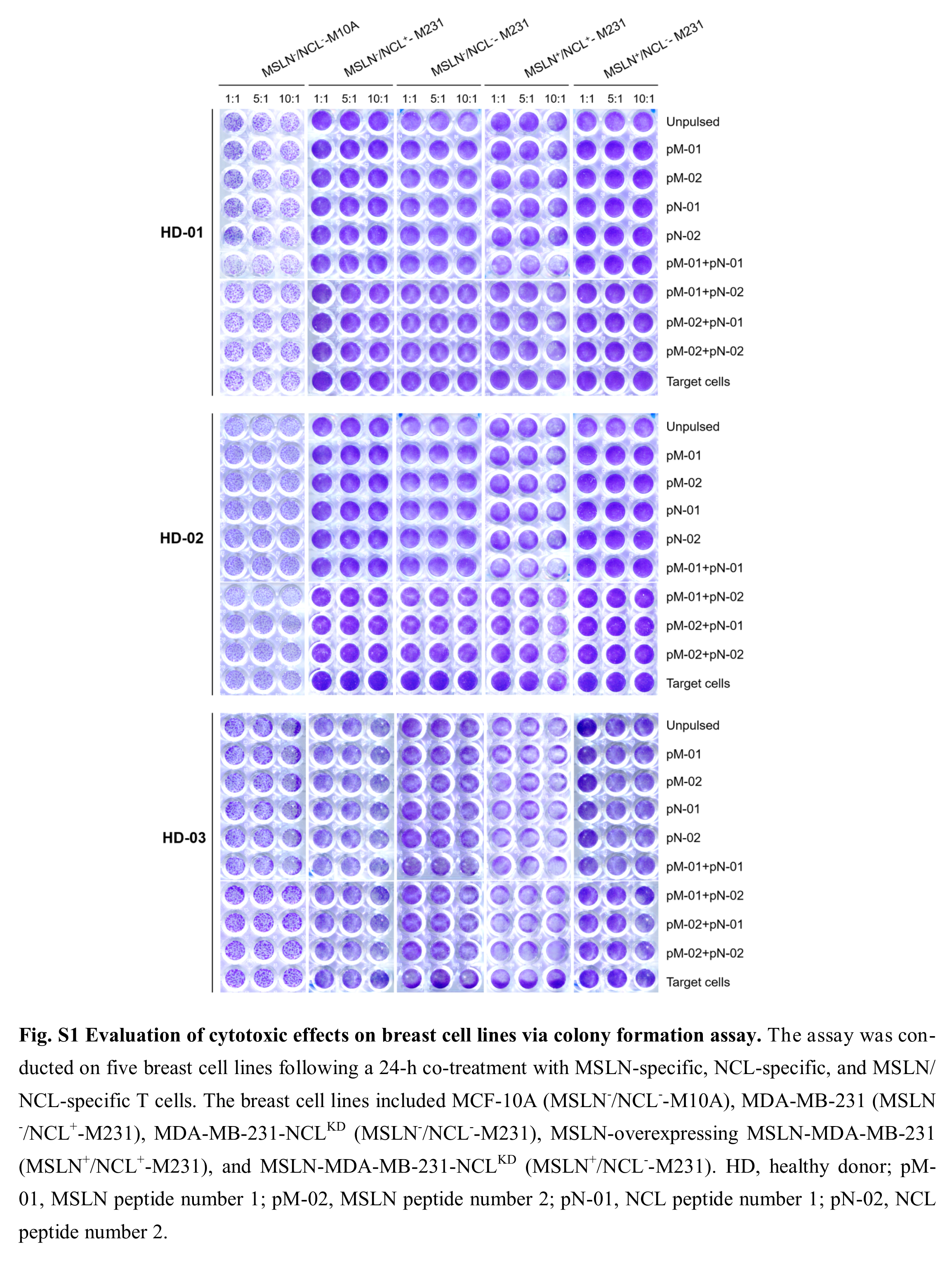

Supplement: Supplementary file 4 — Additional file 4: Figure S1. Evaluation of cytotoxic effects on breast cell lines via colony formation assay. The assay was conducted on five breast cell lines following a 24-h co-treatment with MSLN-specific, NCL-specific, and MSLN/ NCL-specific T cells. The breast cell lines included MCF-10A (MSLN−/NCL−-M10A), MDA-MB-231 (MSLN−/NCL+-M231), MDA-MB-231-NCLKD (MSLN−/NCL−-M231), MSLN-overexpressing MSLN-MDA-MB-231 (MSLN+/NCL+-M231), and MSLN-MDA-MB-231-NCLKD (MSLN+/NCL−-M231). HD, healthy donor; pM-01, MSLN peptide number 1; pM-02, MSLN peptide number 2; pN-01, NCL peptide number 1; pN-02, NCL peptide number 2. [file 12916_2024_3625_MOESM4_ESM.tif]

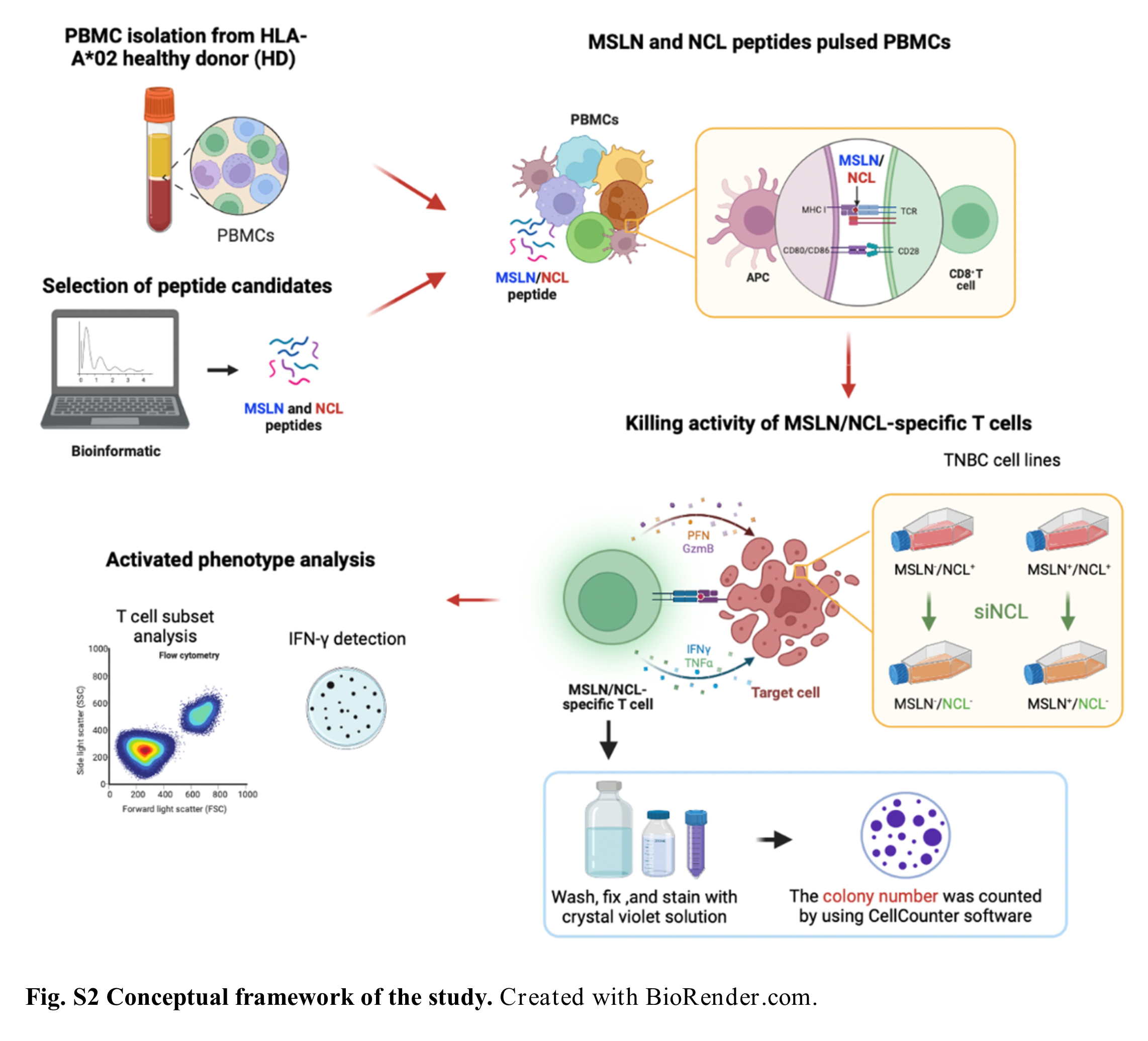

Supplement: Supplementary file 5 — Additional file 5: Figure S2. Conceptual framework of the study. Created with BioRender.com. [file 12916_2024_3625_MOESM5_ESM.tif]

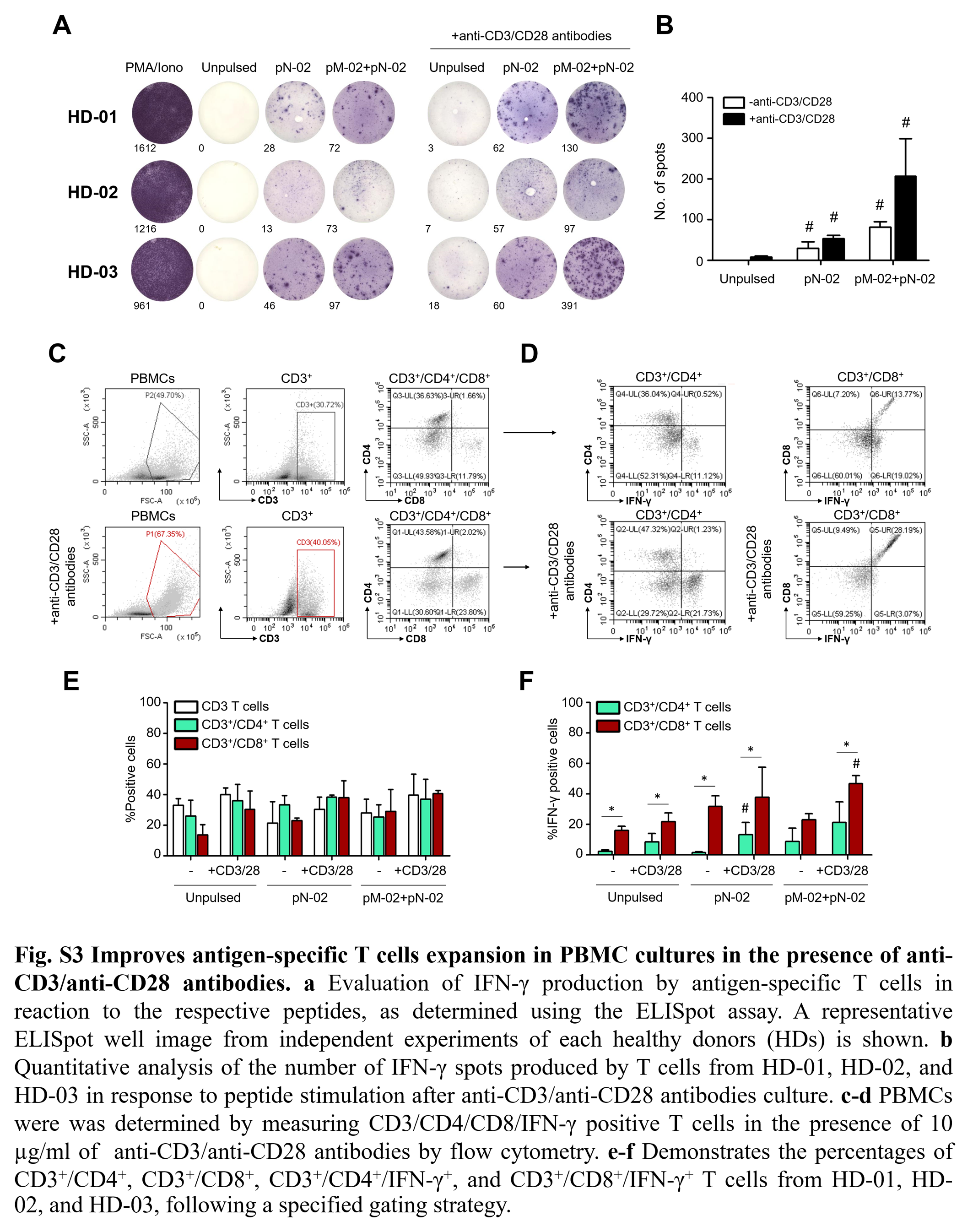

Supplement: Supplementary file 6 — Additional file 6: Figure S3. Improves antigen-specific T cell expansion in PBMC cultures in the presence of anti-CD3/anti-CD28 antibodies. a Evaluation of IFN-γ production by antigen-specific T cells in reaction to the respective peptides, as determined using the ELISpot assay. A representative ELISpot well image from independent experiments of each healthy donor (HD) is shown. b Quantitative analysis of the number of IFN-γ spots produced by T cells from HD-01, HD-02, and HD-03 in response to peptide stimulation after anti-CD3/anti-CD28 antibodies culture. c-d PBMCs were determined by measuring CD3/CD4/CD8/IFN-γ positive T cells in the presence of 10 µg/ml of anti-CD3/anti-CD28 antibodies by flow cytometry. e–f Demonstrates the percentages of CD3+/CD4+, CD3+/CD8+, CD3+/CD4+/IFN-γ+, and CD3+/CD8+/IFN-γ+ T cells from HD-01, HD-02, and HD-03, following a specified gating strategy. [file 12916_2024_3625_MOESM6_ESM.tif]
