# Supplementary material for: Mesothelin- and nucleolin-specific T cells from combined short peptides effectively kill triple-negative breast cancer cells
Source: BMC Med. 2024 Sep 18;22:400. doi: 10.1186/s12916-024-03625-3 (PMC11411782; doi:10.1186/s12916-024-03625-3)
Supplement: Supplementary file 7 — Additional file 7. Uncropped membrane pictures. [file 12916_2024_3625_MOESM7_ESM.pdf]

Figure. 4A

Original western blot for three repeats

Repeat 1

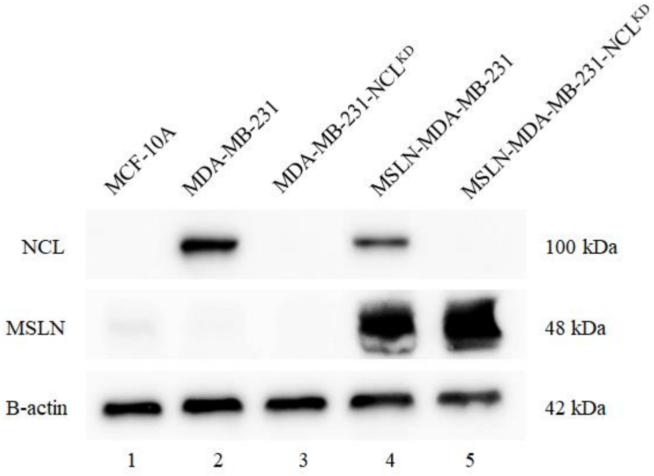

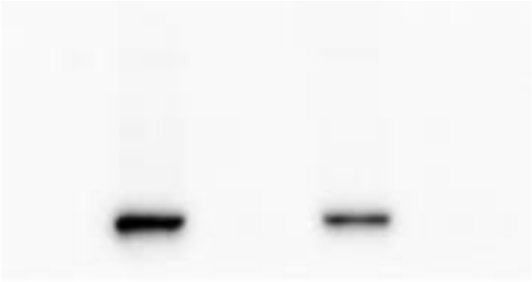

NCL 75 kDa

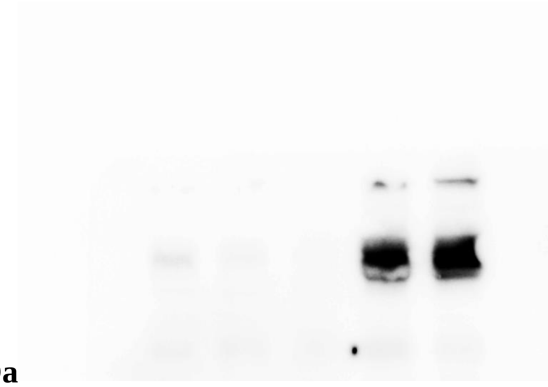

MSLN 48 kDa

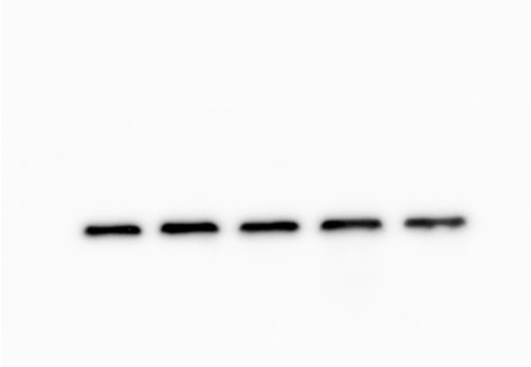

B-actin 42 kDa

Repeat 2

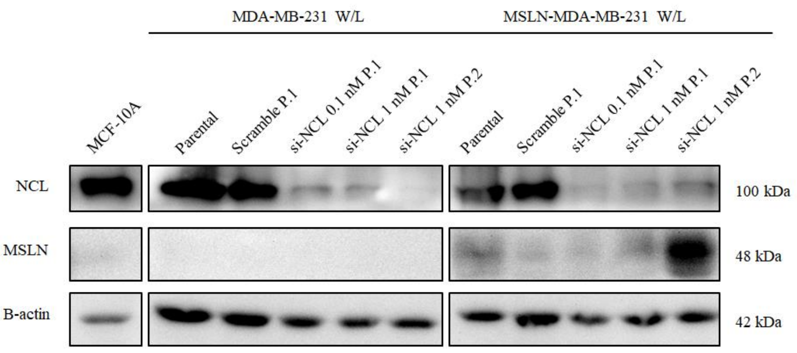

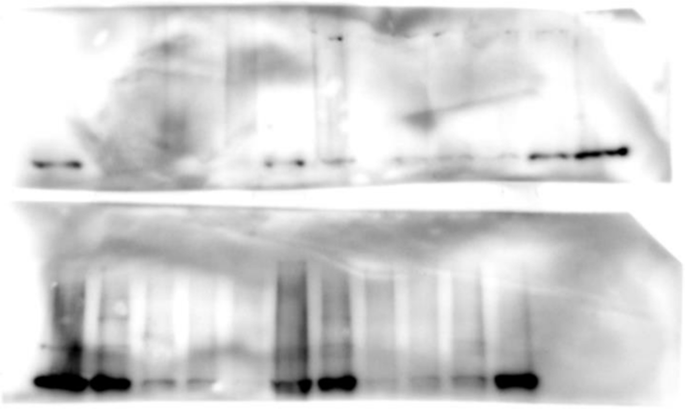

NCL 75 kDa

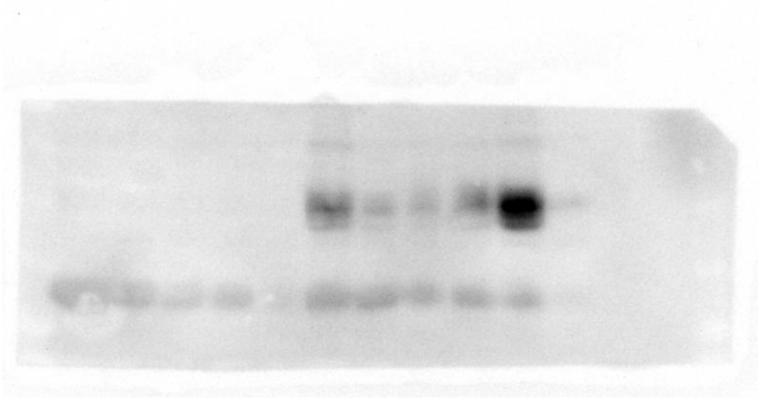

MSLN 48 kDa

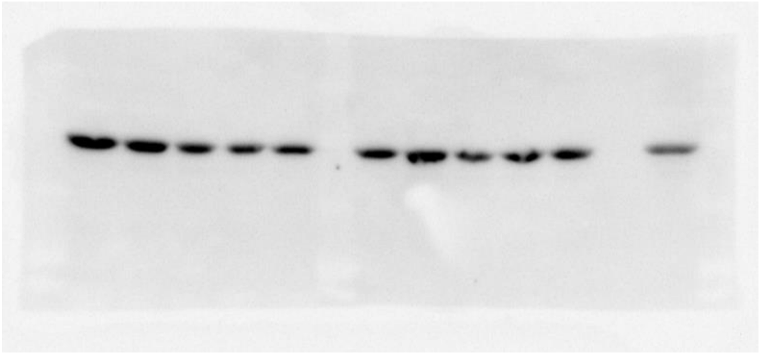

B-actin 42 kDa

Repeat 3

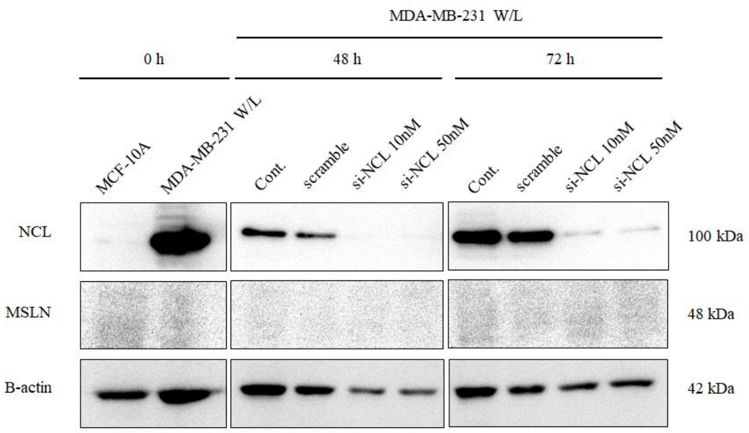

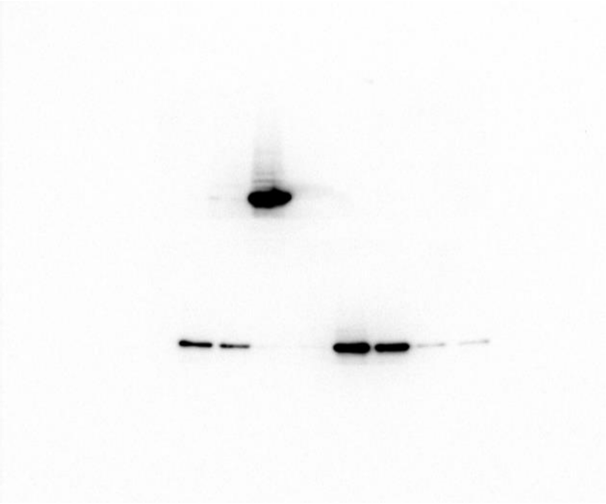

NCL 75 kDa

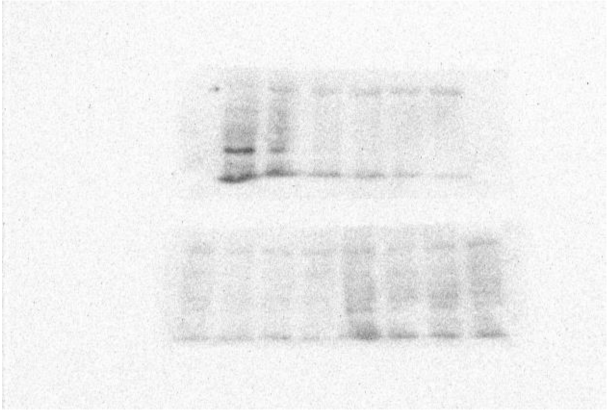

MSLN 48 kDa

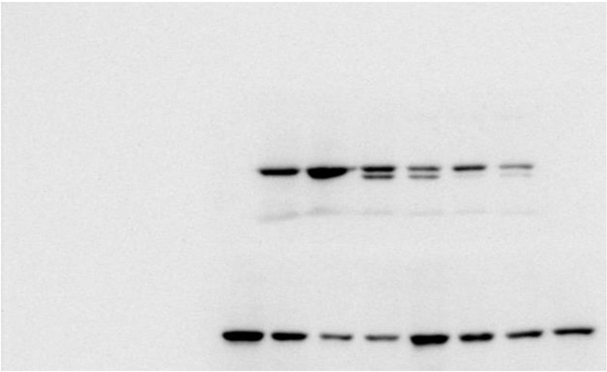

B-actin 42 kDa
